# Optimal Electrode Size for Multi-Scale Extracellular-Potential Recording From Neuronal Assemblies

**DOI:** 10.3389/fnins.2019.00385

**Published:** 2019-04-26

**Authors:** Vijay Viswam, Marie Engelene J. Obien, Felix Franke, Urs Frey, Andreas Hierlemann

**Affiliations:** ^1^Department of Biosystems Science and Engineering, ETH Zurich, Basel, Switzerland; ^2^MaxWell Biosystems AG, Basel, Switzerland

**Keywords:** electrode size, impedance, microelectrode array, extracellular recording, extracellular action potential, local field potential

## Abstract

Advances in microfabrication technology have enabled the production of devices containing arrays of thousands of closely spaced recording electrodes, which afford subcellular resolution of electrical signals in neurons and neuronal networks. Rationalizing the electrode size and configuration in such arrays demands consideration of application-specific requirements and inherent features of the electrodes. Tradeoffs among size, spatial density, sensitivity, noise, attenuation, and other factors are inevitable. Although recording extracellular signals from neurons with planar metal electrodes is fairly well established, the effects of the electrode characteristics on the quality and utility of recorded signals, especially for small, densely packed electrodes, have yet to be fully characterized. Here, we present a combined experimental and computational approach to elucidating how electrode size, and size-dependent parameters, such as impedance, baseline noise, and transmission characteristics, influence recorded neuronal signals. Using arrays containing platinum electrodes of different sizes, we experimentally evaluated the electrode performance in the recording of local field potentials (LFPs) and extracellular action potentials (EAPs) from the following cell preparations: acute brain slices, dissociated cell cultures, and organotypic slice cultures. Moreover, we simulated the potential spatial decay of point-current sources to investigate signal averaging using known signal sources. We demonstrated that the noise and signal attenuation depend more on the electrode impedance than on electrode size, *per se*, especially for electrodes <10 μm in width or diameter to achieve high-spatial-resolution readout. By minimizing electrode impedance of small electrodes (<10 μm) via surface modification, we could maximize the signal-to-noise ratio to electrically visualize the propagation of axonal EAPs and to isolate single-unit spikes. Due to the large amplitude of LFP signals, recording quality was high and nearly independent of electrode size. These findings should be of value in configuring *in vitro* and *in vivo* microelectrode arrays for extracellular recordings with high spatial resolution in various applications.

## Introduction

A current trend in the use of extracellular electrodes for *in vitro* and *in vivo* recordings of neuronal electrical activity (Buzsáki, 2004) is to increase spatio-temporal resolution to capture the dynamics of individual neurons or interactions within neuronal networks (Alivisatos et al., 2013; Marblestone et al., 2013; Rossant et al., 2016; Zeck et al., 2017). High-density multi-electrode arrays (HD-MEAs) can provide long-term, high-resolution activity maps of local field potentials (LFPs) and extracellular action potentials (EAPs) from populations of neurons at the sub-millisecond time scale and spatial scales below 100 μm (Obien et al., 2015).

One way to increase spatial resolution in HD-MEAs is to increase the number of electrodes and, consequently, the number of available readout channels by time-multiplexing multiple electrode signals on only few wires to off-chip circuitry. Such an increase is facilitated by the use of complementary metal-oxide semiconductor (CMOS) technology, which also allows integrating additional circuit components, such as filters, amplifiers, and analog-to-digital converters (ADCs), within a relatively small area on the same substrate as the electrodes. The proximity between electrodes and readout circuitry can also improve signal quality. Indeed, conventional passive MEAs for *in vitro* applications (Thomas et al., 1972; Gross et al., 1977; Pine, 1980; Csicsvari et al., 2003), which typically include just a few metal electrodes with a spatial resolution of typically ≥100 μm, have been supplanted by CMOS-based HD-MEAs in the last decade.

State-of-the-art *in vitro* HD-MEAs integrate tens of thousands of electrodes and feature spatial resolutions of <20 μm with thousands of peripheral recording amplifiers on a single chip (Eversmann et al., 2003; Berdondini et al., 2009; Frey et al., 2010; Du et al., 2011; Hierlemann et al., 2011; Huys et al., 2012; Johnson et al., 2013; Ballini et al., 2014; Bertotti et al., 2014; Rossant et al., 2016; Dragas et al., 2017; Tsai et al., 2017). Similarly, for *in vivo* neural acquisition systems, early devices, such as the stereotrode (McNaughton et al., 1983), tetrode (O’Keefe and Recce, 1993; Gray et al., 1995), and micro-needle probe (Campbell et al., 1991) for recording extracellular field potentials from the intact brain, have yielded way to HD-MEAs of several hundred electrodes microfabricated on a thin silicon shaft (Najafi and Wise, 1986; Blanche, 2005; Wise et al., 2008; Ward et al., 2009; Jun et al., 2017; Mora Lopez et al., 2017), which are now being used in large-scale multi-unit recording systems.

The two factors limiting MEA density are (1) the size and density of the electrodes and (2) the area occupied by the readout circuitry and wiring. The latter depends, in part, on the minimum feature sizes achievable in CMOS technology, which continues to shrink in accordance with Moore’s law (Moore, 2005). The former depends on numerous factors and comprise the primary focus of this article.

A wide range of electrode sizes has been used for extracellular recording. For *in vivo* probes, the sizes range from 10 to 125 μm in diameter (Hubel, 1957; McNaughton et al., 1983; Campbell et al., 1991; O’Keefe and Recce, 1993; Blanche, 2005; Ward et al., 2009; Du et al., 2011; Mora Lopez et al., 2017). For *in vitro* applications, Kim et al. (2014) explored electrode sizes ranging from 5 to 120 μm in diameter. Is there a universally optimal electrode size for extracellular electrophysiology applications, or does the size have to be adapted for detecting desired features, such as axonal signal propagation? Is there a minimum size below which efficient signal detection becomes untenable? A common assumption is that large electrodes (diameter > 50 μm) are well suited for recording population-wide LFPs, while small electrodes (diameter < 20 μm) are more suitable for detecting EAPs from a few nearby neurons (Rossant et al., 2016). We investigate these assumptions in this article.

Other decisive parameters include the overall electrode area and the electrode–electrolyte interface characteristics. Pioneering studies on the electrolyte interface model for extracellular metal microelectrodes by Robinson (1968) established how several electrode properties (e.g., size, material, etc.) can influence the quality of the recorded signals. Since then, there has been extensive research reported on modeling (Buitenweg et al., 2002; Hassibi et al., 2004; Franks et al., 2005; Guo et al., 2012; Thakore et al., 2012), characterizing (Hubel, 1957; Hughes et al., 2000; Camuñas-Mesa and Quiroga, 2013; Spira and Hai, 2013; Ness et al., 2015; Harris et al., 2016; Massobrio et al., 2016), and enhancing the performance of extracellular electrodes (e.g., through novel nanomaterials, structures, and surface chemistry) (Hughes et al., 2000; Ahuja et al., 2008; Ward et al., 2009; Heim et al., 2012; Kim et al., 2014). However, boundary conditions and performance limitations attributable to electrode size have not yet been fully characterized experimentally, especially for electrodes <10 μm in diameter.

In this article, we characterize the impact of electrode miniaturization and spatial density on signal quality and information content within the constraints of the applications under consideration. We considered the following types of extracellular potentials: (1) LFPs from neuronal populations in the frequency range from 1 Hz to 300 Hz and EAPs from individual neurons in the frequency range from 300 Hz to 5 kHz. We then subdivided those EAPs into (2) neuronal EAPs or nEAPs corresponding to the largest detectable EAP of a neuron and (3) axonal EAPs or aEAPs propagating along axons. We characterized the amplitude, spatial spread, and temporal dynamics of a wide range of extracellular potentials from HD-MEA experiments in acute slices, organotypic slice cultures, and dissociated cell cultures.

In order to characterize signal quality, recorded by electrodes, we investigated the effects of signal averaging, “being at the right spot” (i.e., being close to a signal source), and electrode impedance through simulations and experiments. Aside from neuronal signals, we also used a pipette tip as a point-current source and precisely controlled the location of signal source with respect to the electrodes in order to study how potential spatial decay affects signal quality using different electrode sizes. To characterize the noise in both LFP and EAP frequency ranges, we measured the impedance and noise levels of electrodes ranging from 1 μm × 1 μm to 100 μm × 100 μm in size. We summarize the results by providing the estimated signal-to-noise ratio (SNR) for different electrode sizes and applications.

## Materials and Methods

### Ethics Statement

All use of animals and all experimental protocols were approved by the Basel Stadt veterinary office according to Swiss federal laws on animal welfare.

### Measurement Platform

**FIGURE 1:**
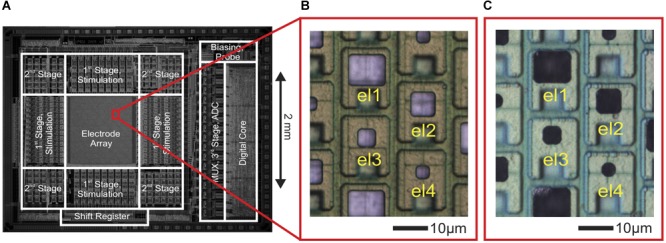
CMOS high-density microelectrode array (HD-MEA) and electrodes of varying size. **(A)** Die micrograph of the CMOS-based microelectrode system (Frey et al., 2009, 2010), fabricated in a 0.6-μm CMOS 3M2P process, that was used for extracellular neuronal recording and stimulation. The electrode array was integrated into a microsystem chip and featured a total of 11,011 electrodes in an area of 1.99 × 1.75 mm^2^. **(B)** Bright Pt electrodes of four different sizes (el1: 10 × 8.6 μm^2^, el2: 6.6 × 6.6 μm^2^, el3: 4.7 × 4.7 μm^2^, el4: 3.3 × 3.3 μm^2^). **(C)** The same electrodes after Pt-black deposition.

We used a CMOS-based high-density MEA (HD-MEA) system (Frey et al., 2009, 2010) for electrode characterization and for conducting extracellular neuronal recordings (Figure 1A). The electrode array was integrated into a microsystem chip and featured a total of 11,011 electrodes in a hexagonal pattern in an area of 1.99 mm × 1.75 mm (18 μm center-to-center pitch, 3’150 electrodes/mm^2^ density). The CMOS microsystem has been fabricated in a 0.6-μm CMOS 3M2P technology, and post-processed at wafer level to (i) produce long-term stable Pt-electrodes and to (ii) further enhance the passivation layer to protect the circuitry against culturing media. The post-processing steps have been described before (Frey et al., 2009, 2010). In brief, Si_3_N_4_ was first deposited by means of plasma-enhanced chemical vapor deposition (PECVD), and the pads and electrodes were subsequently re-opened through reactive-ion etching (RIE). Next, TiW (50 nm), for promotion of the adhesion, and Pt (270 nm) as electrode material were ion-beam-deposited and then patterned by using an ion-beam etching step. A 4-layer 1.6-μm-thick passivation stack, consisting of alternating SiO_2_ and Si_3_N_4_ layers was deposited by PECVD; finally, a re-opening of the platinum electrodes was achieved through an RIE step. The Pt-metal layer in the electrode openings is free of features to ensure good connectivity and adhesion of a post-processed Pt-layer.

Up to 126 electrodes could be simultaneously recorded by connecting the electrodes to read-out channels through a flexible switch matrix underneath the electrode array. The switch-matrix approach provided low-noise voltage recordings and large routing flexibility to select almost arbitrary electrode configurations to connect to the readout circuits. On-chip circuitry was used to amplify (0–80 dB programmable gain), filter (high pass: 0.3–100 Hz, low pass: 3.5–14 kHz), and digitalize (8 bit, 20 kSps) the recorded signals, which were then sent to a field-programmable gate array (FPGA) board. Finally, the data were streamed to a host PC for data storage and real-time visualization. Data analysis was performed by using MATLAB R2014b (The Mathworks). Another CMOS-based HD-MEA (Ballini et al., 2014; Müller et al., 2015), with a regular-grid-electrode-array format (same CMOS post-processing), was used to investigate the characteristics of “pseudo-large” electrodes through a local combination of several electrodes, the signals of which then were routed to the same amplifier channel.

### Multi-Size Electrode Fabrication and Pt-Black Deposition

#### Active Electrode Arrays on CMOS Chips

As shown in Figure 1B, Pt-electrodes of four different sizes (el1: 10 μm × 8.6 μm, el2: 6.6 μm × 6.6 μm, el3: 4.7 μm × 4.7 μm, el4: 3.3 μm × 3.3 μm) were fabricated on the CMOS-based HD-MEA through wafer-level post-processing as described above to characterize the electrode noise and to subsequently perform electrophysiology recordings.

#### Passive Electrode Arrays on Silicon Chips

For detailed electrode–electrolyte impedance spectroscopy measurement and estimation of the electrode thermal noise, a wide variety of Pt-electrodes were fabricated on silicon substrates with sizes ranging from 100 μm × 100 μm down to 1 μm × 1 μm. Four-inch silicon (100) wafers (Wacker Chemie, Burghausen, Germany) were used as substrates. First, a two-layer photoresist was spun on and subsequently processed to form the lift-off masking layer. A metal stack consisting of a 20 nm WTi10 alloy adhesion layer and a 200 nm platinum layer was deposited by ion beam deposition. With the subsequent lift-off solvent soak and cleaning steps the metal patterns were unveiled, which formed the electrodes and leads. A 0.2-μm-thick silicon oxide and a 1.4-μm-thick silicon nitride layer were then deposited for passivation. The platinum electrodes and contact pads were opened through an RIE step. The contact pads of the fabricated dies were then wire-bonded to a 64-pad PCB to access each electrode.

For reducing electrode impedance, for both passive and active electrode arrays, Pt-black was electrochemically deposited on the electrodes (Figure 1C). A current of 1 nA/μm^2^ was simultaneously applied to all electrodes for 45–75 s while using a platinum wire as a ground electrode, immersed in the deposition solution [0.7 mM hexachloroplatinic acid and 0.3 mM lead (II) acetate anhydrous]. For details, see Frey et al. (2009, 2010).

### Impedance Measurements

Impedance measurements were performed in phosphate-buffered saline solution (PBS, same electrical properties as the medium used for neural culturing and plating). For impedance spectroscopy, a commercial potentiostat (Ivium CompactStat, Eindhoven, Netherlands), equipped with a frequency response analyzer (FRA module) was used. Measurements were performed between 1 Hz and 100 kHz with an alternating voltage amplitude of 10 mV peak to peak. Four points per frequency decade were recorded. The applied working potential during the measurements was maintained at 0.3 V against an Ag/AgCl reference electrode. Two-point impedance measurements were performed by using a lock-in amplifier (Zurich Instruments, Zurich, Switzerland) to verify the recordings with the Ivium potentiostat.

### Noise Measurements

**FIGURE 2:**
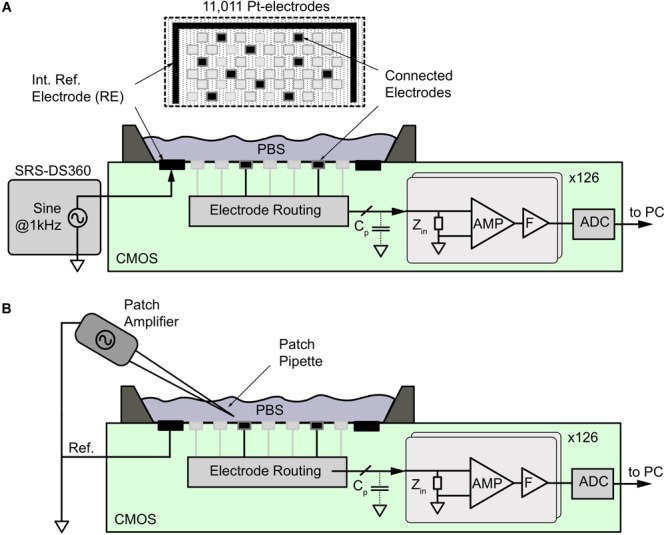
Noise and signal-attenuation measurements of electrodes by using the CMOS HD-MEA. **(A)** For signal attenuation measurements, a sinusoidal signal at 1 kHz from a DS360 signal generator was applied to the phosphate buffer solution (PBS) through an internal reference electrode (RE), while the signal was acquired from different electrode types within the 11,011-electrode array as shown at the top. For noise measurements, the reference electrode was disconnected from the signal source, and a fixed potential was applied. The electrode-electrode interface noise was then amplified (AMP), filtered (F), and digitized (ADC) by using the internal voltage recording channels. **(B)** A Patch amplifier and pipette was used to generate point-source signals to evaluate the averaging effect of the electrodes of different sizes.

To measure the intrinsic noise level of the electrodes, on-chip circuitry in the HD-MEA (Frey et al., 2010) was used. As shown in Figure 2A, all microelectrodes were placed in the same physiological liquid at 37°C. A 500-μm-thick Pt-wire of 10 mm length was used as a reference electrode. The electrical potential across the microelectrodes was recorded for 100 s to obtain the integrated noise down to the sub-Hertz frequency range. Signals were amplified (960×), and low-pass filtered around 5 kHz by using on-chip active filters. Data were sampled at 20 kHz and digitized using the on-chip ADCs and analyzed by using MATLAB 2014b oﬄine. For the integrated noise values, the standard deviation of the signal (σ_s_) was calculated for the EAP and LFP band during a 100-s recording period for each electrode of the array. As this noise included the intrinsic noise of the electrodes (σ_el_) and the electronic noise of the amplifiers (σ_a_), we estimated the intrinsic noise level of each electrode under the assumption of statistical independence of these two noise contributions as:

σ_el_^2^ = σ_s_^2^ – σ_a_^2^

The input-referred noise (σ_a_) of the amplifier was measured separately without connecting the electrodes.

### Signal-Attenuation Measurements

To determine the signal-attenuation of the electrode, the amplifier interface was measured by using the CMOS HD-MEA system. As shown in Figure 2A, an alternating sinusoidal voltage at 1 kHz with an amplitude of 1 mV (V_stim_) peak-to-peak was applied to the reference electrode (RE) on the HD-MEA chip in the PBS solution by means of an external function generator (DS360 ultra-low distortion function generator, Stanford Research Systems, Sunnyvale, CA, United States). The signals recorded at the working electrode (more than 30 electrodes per size) were attenuated through the electrode impedance (*Z*_el_), the amplifier input impedance (*Z*_a_), and the parasitic shunt capacitance (*C*_p_) in the path between the electrode and the amplifier. The signals were amplified (960×), band-pass filtered (1 Hz–5 kHz), digitized using on-chip ADCs and then sent to a PC. The acquired signals were extracted oﬄine in MATLAB to obtain the input-referred signal amplitude (*V*_meas_) at 1 kHz. The signal-attenuation was then calculated from *V*_meas_.

### Model of a Point-Current-Source on the HD-MEA

The mathematical model used here was adapted from Obien et al. (2019). The model assumed a point-current source located above an HD-MEA in a homogeneous and isotropic extracellular medium. As a boundary condition, the electrode surface was considered to be an infinite insulating plate (Ness et al., 2015). To solve for the scalar potential that a current source I above the insulating plate generates, the method of images (MoI) was applied. The MoI includes considering another identical current source, I, on the opposite side of the *x*–*y* plane of the insulating plate. This method enables the scalar potential to be solved by using the contributions from both current sources with no insulator present. The model is described by:

(1) $$\begin{array}{c} V=I/(2\pi \sigma \sqrt{X^{2}{+Y}^{2}{+Z}^{2}}) \end{array}$$

where I is the current; σ is the medium conductivity; *X* = (*x* – *x*′), *Y* = (*y* – *y*′) and *Z* = (*z* – *z*′). *V* is the signal amplitude at location (*x*′, y′, z′), where *z*′ = 0, as the array surface is flat; (*x, y, z*) are the coordinates of the point-current source. The model was implemented in MATLAB, and signal amplitudes at locations (*x*′, *y*′) = ([0–20 μm], [0–20 μm]) were obtained for source positions at (*x, y*) = 0, *z* = [1, 7, 10, 20, 100 μm]. We modeled the spatial-averaging effect as a consequence of the electrode size by modeling V at 100 random points within an electrode area. The voltage values at those 100 points were then averaged to obtain the V-value for the simulated electrode.

### Point-Current-Source Recordings Through Micropipette Stimulations

Borosilicate glass micropipettes with filaments were pulled using a P-97 pipette puller (Sutter Instruments) to have sharp tips with tip resistances of 7–13 MΩ. The tip resistance and output current were monitored with the Clampex software (Molecular Devices, Sunnyvale, CA, United States). A glass micropipette was filled with Ca-free artificial cerebrospinal fluid (ACSF, contents in mM: NaCl 125, KCl 2.5, NaH_2_PO_4_ 1.25, MgSO_4_ 1.9, glucose 20, NaHCO_3_ 25), connected to a patch amplifier (MultiClamp 700B, Molecular Devices, Sunnyvale, CA, United States), and mounted on a micromanipulator (Patch-star, Scientifica, East Sussex, United Kingdom). The stimulation signal was digitally generated and then imported to Clampex. The patch amplifier controlled the stimulation amplitude. The pipette was positioned atop the HD-MEA (Figure 2B) by using the micromanipulator, and its distance from the array surface was determined using a microscope (Olympus BX61 with a 40× water immersion objective). The chamber atop the HD-MEA was also filled with ACSF. The micropipette was set to have a square-wave peak-to-peak output current of 50 nA at 1 kHz. An external Ag/AgCl pellet was used as a reference electrode and was placed in the HD-MEA chamber with ACSF. This reference electrode was connected to the external reference node of the HD-MEA together with the reference of the micropipette. The square-wave signal from the micropipette mimicked a point-current source. HD-MEA recordings captured the spatial-spread of the point-current-source signal. The signal amplitude detected by each electrode was extracted using a demodulation script in MATLAB.

### Spatial-Averaging by Electrode Size: Point-Current Source

To investigate the spatial-averaging of the signals at the micrometer scale, we recorded the signal from a micropipette (square-wave stimulation, 1,000 Hz, 50 nA) at 7 μm distance above an electrode and moved it laterally, up to 20 μm, in parallel to the HD-MEA surface (Figure 2B). We measured the signal from one electrode and repeated the same procedure for different electrode sizes. In this way, we were able to obtain the signal amplitude from a point-current source at distances below the pitch of the HD-MEA electrodes. The spatial-averaging effect was also simulated for a planar array (Supplementary Figure S2), using Eq. (1).

### Electrode “Being at the Right Spot”

The effect of electrode density on the recorded signal was quantified using MATLAB. First, we modeled a dense-array of point electrodes arranged at different electrode pitches (i.e., 1, 2, 4, 8, 16, 32, 64, 100 μm). Then, we placed 100 point-current-sources (as a neuronal model) randomly over the array area (1 mm × 1 mm) at a fixed *z*-distance of 7 μm. The signal amplitudes, measured by the electrodes, depended on the distance (*d*′ = √(*x*^′2^+ *y*^′2^+ *z*^′2^) between the electrodes and each of these point-current-sources (located at *x*′*y*′*z*′) and were computed using Eq. (1).

The average distance of a point-source from the electrode relative to the electrode pitch was computed numerically. Calculation details are shown in the Supplementary Figure S4. Here, we considered electrodes in grids as well as hexagonal arrangements, both for a *z*-distance of zero. In the grid arrangement, the average distance *d*^G′^_avg_ was calculated by using the model of a point in a square as shown in Supplementary Figure S4a. The model of a point in a triangle was used to calculate the average distance *d*^H′^_avg_ for electrodes in a hexagonal arrangement. The effect of electrode density or “being at the right spot” was then plotted (see Supplementary Figure S5) for different *z*-distances using the calculated *d*^G′^_avg._ or *d*^H′^_avg_ parameters.

(2) $$\begin{array}{c} V=I/(2\pi \sigma \sqrt{({\left(d_{\mathrm{avg}}^{G}\right)}^{2}{+Z}^{2}})) \end{array}$$

The value used for the current (I) was 1 pA; the medium conductivity (σ) was defined to be 0.3 S.

### Cortical Cell Cultures

Primary cell cultures were prepared as described in Bakkum et al. (2013), in accordance with Swiss Federal Laws on animal welfare. Briefly, cells from embryonic day 18 wistar rat cortices were dissociated in 2 ml of trypsin with 0.25% EDTA (Invitrogen, CA, United States) with trituration. The electrode array surface was pre-coated with a thin layer of poly(ethyleneimine) (Sigma, MO, United States), 0.05% by weight in borate buffer (Chemie Brunschwig, Basel, Switzerland) at 8.5 pH, followed by a 10 ml drop of 0.02 mg/ml laminin (Sigma) in Neurobasal medium (Invitrogen, CA, United States) for cell adhesion. 20,000–30,000 cells in a 6-μl drop were seeded over the array, and 1 ml of plating medium was added after 30 min. After 24 h, the plating medium was changed to the growth medium. Plating medium consisted of 850 ml of Neurobasal, supplemented with 10% horse serum (HyClone, UT, United States), 0.5 mM GlutaMAX (Invitrogen, CA, United States) and 2% B27 (Invitrogen, CA, United States). Growth media consisted of 850 ml of DMEM – Dulbecco’s Modified Eagle Medium (Invitrogen, CA, United States), supplemented with 10% horse serum, 0.5 mM GlutaMAX and 1 mM sodium pyruvate (Invitrogen, CA, United States). The cultures were maintained inside an incubator to control environmental conditions (37C, 65% humidity, 9% O_2_, 5% CO_2_) in 1 ml of growth medium (partially replaced twice per week).

### Organotypic Slice Cultures

Organotypic hippocampal cultures were prepared as described in Gong et al. (2016). To obtain brain slice cultures, newborn Thy1-YFP mice at postnatal days 5–7 were used. Under sterile conditions, brains were removed and placed in ice-cold, oxygenated (95% O_2_ + 5% CO_2_) HBSS (HANK’S balanced salt solution, GIBCO 14175). Bi-lateral hippocampi were dissected and embedded in low-melting-temperature agarose solution (1%, Sigma-Aldrich, A9414). A vibratome (Leica VT1200 S) was used to obtain 300-μm-thick sagittal hippocampal slices. The slices were attached on the HD-MEA surface by using a mixture of chicken plasma (500 U/ml Sigma-Aldrich P3266) and thrombin from bovine plasma (200 U/ml, Sigma-Aldrich T4648). Before slice attachment, the HD-MEA chip was sterilized in 70% ethanol for 40 min and coated with 0.05% PEI (polyethyleneimine, pH = 8.5, Sigma-Aldrich; Bakkum et al., 2013). After the slice had been placed on the HD-MEA surface, a culture medium (3 ml, contained basal medium eagle without L-glutamine, Hanks’ balanced salt solution, inactivated horse serum, 45% D-glucose, GlutaMAX, with/without penicillin-streptomycin, and with/without B27 supplement) was supplied. The hippocampal slices were cultivated in culture chambers, which were kept rotating on a rotation rack. The culture chambers were placed inside an incubator with controlled temperature (36°C), humidity (90%) and CO_2_ (5%). After 3 days, the culture medium was replaced with culture medium containing penicillin streptomycin without B27. The details of the procedure have been described in previous papers (Gähwiler, 1981; Van Bergen et al., 2003).

### Acute Brain Slices

Adolescent wild-type CD-1 mice (P18 to P23) were deeply anesthetized by isoflurane inhalation and then decapitated. The dissected brains were immediately immersed in ice-cold dissection ACSF. Cerebellar and cortical tissues were obtained. The cerebellum was glued onto the vibratome tray along its sagittal plane, and the cortex along its coronal plane. The tissues were kept in ice-cold dissection ACSF bubbled with carbogen (95% O_2_ and 5% CO_2_) during slicing. Parasagittal cerebellar slices and coronal cortical slices (200 μm thick) were cut using a Leica VT-1200S vibratome. The slices were transferred to carbogen-bubbled warm ACSF (35°C) and were allowed to recover in incubation for at least 40 min before placement on the HD-MEA. The other slices were maintained at room temperature until measurement. Slices were carefully positioned flat on the HD-MEA surface for recording. Slices were kept in place by using a weight that has been custom-made by attaching a transparent membrane onto a small platinum ring. A hole was cut on the membrane so that a micropipette could penetrate deep into the slice. Slices were superfused with carbogen-bubbled recording ACSF at 36°C. Spontaneous activity detected by the HD-MEA in this setup persisted up to 8 h after incubation. Cortical slices in adjusted ACSF (1 mM Mg^2+^ and 1 mM Ca^2+^) exhibited both LFPs and EAPs across different cortical layers in HD-MEA recordings.

### HD-MEA Extracellular Recordings

For dissociated-cell and organotypic-slice cultures, the HD-MEA recording setup was placed in a recording incubator (65% humidity) for control of the environmental conditions (36°C and 5% CO_2_). During experiments, the HD-MEA devices were transferred to the recording incubator and covered with sterilized PDMS caps to minimize media evaporation. Cultures were allowed to mature for 2–3 weeks before recording.

For acute slices, the HD-MEA recording setup was at room temperature, and the acute tissue was continuously superfused with carbogen-bubbled recording ACSF at 36°C to maintain cell viability.

All LFP+EAP recordings were done at 20 kHz sampling rate, 1–3,700 Hz band-pass filter, and 960× amplification.

### Data Analysis

#### Extraction of Spatio-Temporal Features of EAPs

We recorded spontaneous activity of neurons from different preparations: acute cerebellar brain slices, organotypic hippocampal slice cultures, and primary cortical cell cultures. To identify EAPs, we filtered the raw signals using a band-pass filter (100–3,000 Hz). To extract EAPs from single neurons, we identified electrodes with the largest EAPs across the electrode array and scanned the full array while keeping the selected electrodes in all recordings. We then detected EAP spikes from the selected electrodes using a high threshold (8× RMS noise) and employed template matching (Franke et al., 2015) to sort out the outlier spikes. To extract the spatio-temporal features of EAPs from individual neurons, we used spike-triggered-averaging (Bakkum et al., 2013, 2018; Radivojevic et al., 2016). The timing of the detected spikes per selected electrode was used to average voltage traces on each of the remaining recorded electrodes. We aimed for neurons with a high firing rate in order to average 500 voltage traces per electrode and to achieve high-quality EAP maps per neuron.

#### Extraction of Spatio-Temporal Features of LFPs

We recorded spontaneous LFPs from organotypic hippocampal slice cultures using a grid configuration of electrodes (40 μm pitch). To obtain LFP events, we band-pass filtered the raw traces (1–10 Hz). We first selected the electrodes with possible LFP signals by means of thresholding (>50 μV). We then obtained the mean of the voltage traces across all selected electrodes. Using a threshold of 3× RMS noise on the mean signal, we identified the timing of large LFP events. A negative or positive signal deflection of more than 10 ms duration was considered an LFP event.

#### Spatial-Averaging Through Electrode Size

We analyzed the spatial-averaging effect experimentally by using two methods. First, we used an electrode array with different electrode sizes (el1, el2, el3, and el4) to quantify the spatial-averaging effect for small electrodes. Each electrode in that array (hexagonal arrangement) featured a defined size and was surrounded by a total of six electrodes, i.e., two electrodes of each other size (see Figure 1B,C). We identified a ‘center electrode’ per neuron, which detected the largest spikes for that neuron. Then, we obtained the relative amplitude of the spikes detected from the six neighboring electrodes (hexagonal arrangement) with respect to the largest spike at the center electrode. To estimate the spatial averaging at the center electrode, we computed the slope of the best fit line of the amplitudes versus distance from the location of the peak amplitude. The steepness of the slope indicates the extent of averaging: a steeper slope indicates less of an averaging effect.

The second method, which was used to approximate the spatial-averaging on large electrodes, was applied to EAP and LFP spatio-temporal maps. For EAPs, we selected areas of the EAP spatio-temporal map that corresponded to perisomatic areas (large negative biphasic spikes), dendritic areas (positive spikes during the EAP spike), and axonal branches (triphasic spikes). We then selected the ‘center electrode,’ as an electrode where the largest spike amplitudes were detected. To estimate signals upon using large electrode sizes, we calculated mean spike waveforms by averaging the signals of a set of several neighboring electrodes. The set always included a center electrode and its surrounding electrodes (i.e., 4, 9, 16, and 25 electrodes). Then, we estimated the difference between the spike amplitude obtained with the ‘center electrode’ only and the mean spike waveforms obtained by averaging the electrode sets (4, 9, 16, and 25 electrodes).

#### Signal-to-Noise Ratio (SNR)

To estimate the SNR, the dependence of three main parameters on electrode size were taken into consideration: the spatial-averaging effect; signal-attenuation due to the impedance ratio (*Z*_el_:*Z*_a_); and the noise – both thermal noise of electrode and “background activity.”

(3) $$\begin{array}{c} SNR=\frac{\mu (S_{EAP|LFP})\times \beta_{A}\times \beta_{D}\times \beta_{Z}}{\sqrt{\sigma_{\mathrm{el}}^{2}{+\sigma }_{\mathrm{bg}}^{2}}} \end{array}$$

Here, μ(S_EAPjLFP_) is the mean signal amplitude (EAP and LFP), obtained from the individual neurons that have been identified in the recordings. The parameter β_A_ represents the spatial-averaging effect. The spatial averaging was found to be different in each preparation and for each signal type, so we used the slopes of the best fits for the averaging effect in dependence of the electrode size (for axonal branches, | slope| = 0.04 μm^-1^; somatic areas, | slope| = 0.015 μm^-1^; dendritic areas, | slope| = 0.01 μm^-1^ and for LFPs, | slope| < 0.005 μm^-1^) to estimate the SNRs. The parameter β_D_ represents the effect of electrode density, i.e., the electrode “being at the right spot.” β_z_ is the signal-attenuation parameter and depends on the impedance ratio (*Z*_el_:*Z*_a_). σ_el_ is the thermal noise level of an electrode, which depends on its effective surface area. For the SNR estimation, we considered different levels of background activity (σ_bg_) according to different fractions of active neurons.

## Results

**FIGURE 3:**
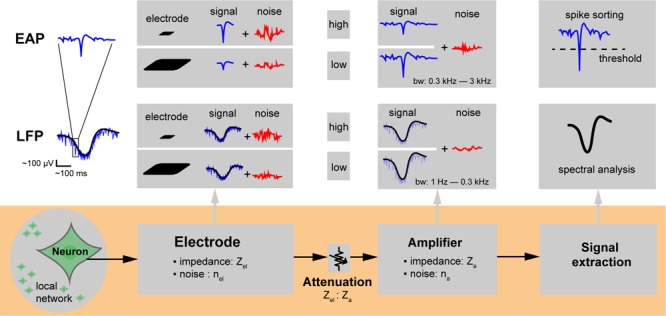
Extracellular recording chain. Microelectrodes for extracellular action potential (EAP) and local field potential (LFP) signal recording. The neuronal signals of interest are attenuated along the overall recording chain and may be compromised through injected noise or interfering signals until they get digitized and stored for analysis. The electrode size affects the spatial signal averaging, which determines and the capability to resolve local features of specific neuronal cells. The impedance of the electrode (*Z*_el_) depends mainly on its overall surface area and material and determines the electrode noise (*n*_el_); the ratio of electrode to amplifier input impedance (*Z*_el_: *Z*_a_) directly affects signal attenuation. Background neuronal activity or undesired EAPs from distant neuronal sources (local network) give rise to noise. The size of the electrodes also determines how much of the background activity is picked up.

Studying the effect of electrode size in extracellular-potential recordings included several essential questions. Which electrode size is best for which application? What is the major contributor to the quality of extracellularly recorded signals? Are smaller electrodes better to resolve details of extracellular-field distributions? To approach this issue, we first considered the overall signal acquisition chain and process on a single electrode. Figure 3 shows the extracellular recording chain including signal and noise sources. Three main components influence the recording quality of extracellular potentials with single electrodes: the characteristics of (1) the neuronal preparation and cell type, (2) the recording electrode, and (3) the readout hardware. The neuronal signals of interest are attenuated along the overall recording chain and will be compromised by noise until they get digitized and stored for analysis. We will first describe the general spatial and temporal characteristics of extracellular potentials from different sources. We will then describe how electrode and array characteristics affect the obtained signal (spatial averaging and location of the electrode at the right spot). Afterward, we will describe the effects of signal attenuation through the readout circuitry and noise sources. Finally, we will summarize the SNR performance of electrodes of different sizes in measuring various types of extracellular potentials.

### Signal Characteristics in Different Preparations

**FIGURE 4:**
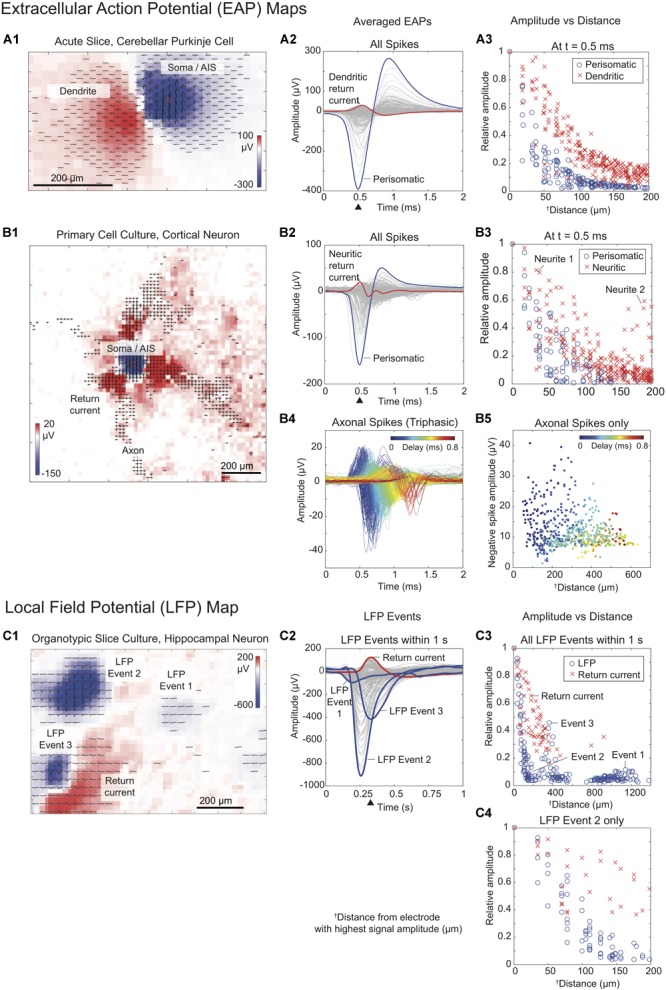
Signal sources. Extracellular action potentials (EAPs) generated by **(A)** a cerebellar Purkinje neuron from an acute-slice recording and **(B)** a cortical neuron from a dissociated cell culture recording. **(A1,B1)** Spatio-temporal features of extracellular action potentials (band-pass filter: 100–3000 Hz). Waveforms were averaged over 500 individual spikes. **(A2,B2)** Averaged EAP spike waveforms. Waveforms in blue indicate the signal with the largest negative spike amplitude (from electrodes marked with red squares in figure **(A1,B1)**, while the waveforms in red show the return current signal with the largest positive amplitude during the spiking events. **(A3,B3)** Relative amplitudes of signals versus distance of the recording electrodes from the location of the electrode with the largest negative amplitude (normalization to the largest negative and positive signal amplitudes). Blue circles refer to the amplitude of the negative spikes originating from the perisomatic area (soma, axon initial segment or AIS). The amplitudes of the return currents during the EAP are shown as red x’s. **(B4)** Axonal spikes, comprising all triphasic spikes in **(B2)** detected after the occurrence of the largest negative spike. **(B5)** Amplitude of the largest negative signal of all axonal signals versus distance of the electrodes from the location of the electrode with the highest-amplitude negative spike. **(C)** Local field potentials recorded from an organotypic hippocampal slice culture. **(C1)** Spatio-temporal features of LFP events that occurred within 1 second at the CA3 and dentate gyrus areas of a hippocampal slice culture (band-pass filter: 1–10 Hz). **(C2)** LFP event waveforms and return currents. The largest return current signal is in red, while the three largest negative peaks indicating the LFP events at different time points are displayed in blue. **(C3,C4)** Relative amplitudes of recorded signals during all LFP events and during LFP event 2 (blue circles) and return current signals (red x’s) versus distance of the respective recording electrodes from the electrode featuring the highest-amplitude signals (normalization to the largest negative and positive signal amplitudes). The black triangles in **(A2,B2,C2)** mark the time points used for electrical mapping of the signal amplitudes recorded from all electrodes shown in **(A1,B1,C1)** (color scale: blue to red).

In order to understand the spatiotemporal features of neural activity, we recorded extracellular potentials from dissociated cell cultures, acute brain slices, and organotypic brain slices using HD-MEAs. We used cortical, hippocampal, and cerebellar samples to explore the extracellular-potential features across different cell types. Figure 4 shows representative examples of extracellular signals and potential distributions for LFPs, nEAPs, and aEAPs.

We first characterized EAPs of cultured neurons. The features of EAPs of individual neurons provide insights into the morphology of the neuron. We observed negative and positive spike waveforms that were monophasic, biphasic, and triphasic, in accordance with what has been reported before (Nam and Wheeler, 2011). Based on previous findings (Frey et al., 2009; Bakkum et al., 2013, 2018; Radivojevic et al., 2016), we could assign and differentiate subcellular compartments according to characteristic waveforms that they produced. The largest negative-amplitude spike was usually found in the perisomatic area (Figure 4A1,A2,B1,B2), in the region of the axonal initial segment (AIS) (Bakkum et al., 2018). This large negative spike is what we consider an nEAP. On the other hand, positive spikes occurring simultaneously with the most negative spike were indicative of return currents that occurred in the regions of dendrites/neurites of the same neuron. In cortical cell cultures, the peak amplitude values of the nEAP range from 0.02 to 1.7 mV. The potential distributions in cell cultures are more localized compared to those in acute brain slices—they fall off quickly at 20 up to 100 μm radius from the peak (20% of the peak), which is a consequence of the fact that cell cultures do not form a dense tissue layer on the electrodes as compared to slices and are typically within very close proximity of the electrodes (the potentials of Purkinje cells in acute slices feature nEAP amplitudes in the range between 20 and 500 μV, are often more distant to the electrodes and fall off within a radius of 50–150 μm from the peak). Triphasic spikes in cortical cell cultures indicated action potentials propagating along axonal branches (Figure 4B1,B4) or aEAPs. The aEAPs were found to be smaller, ranging from 1 to 50 μV amplitude, and were very localized within 20 to 30 μm along the axonal-arbor structure. The duration of monophasic, biphasic, and triphasic EAPs is between 0.5 and 2 ms. The propagation speed of aEAPs ranges between 0.3 and 1 m/s (Bakkum et al., 2013; Radivojevic et al., 2017).

To characterize LFPs, we extracted the spatiotemporal features of single LFP events from different regions in organotypic hippocampal slices (Figure 4C1,C2) and acute cortical slices (Supplementary Figure S1b). The amplitudes of LFPs were comparably large, ranging from 0.1 to 1.5 mV. An LFP can be positive or negative, where the positive signals are mostly attributed to synaptic projections (Shein-Idelson et al., 2017), while negative potentials usually occur during neuronal-population activity, such as network bursts (Buzsáki et al., 2012). The spatial extension of an LFP event ranges between a few hundred micrometers and several millimeters. Moreover, LFPs may propagate depending on the origin of the signal. An LFP event lasts a few hundred milliseconds in brain slices and is shorter (tens of milliseconds) in cell cultures. The presence of LFPs indicates network connectivity. Moreover, LFPs are often used to study oscillations and seizures (Buzsáki et al., 2012; Ray, 2015).

These results show that the spatiotemporal features of extracellular potentials are different for every preparation. The electrode size may influence the detection of EAP or LFP signals and, therefore, the subsequent data analysis and identification of signal sources. It is important to know which electrode size is best for which specific recording scenario to obtain all relevant information.

### Spatial Averaging due to Electrode Size

**FIGURE 5:**
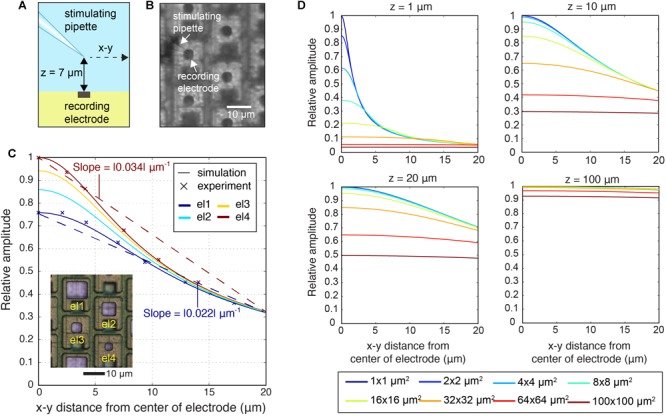
Spatial-averaging of a point-source signal by the electrodes. **(A)** Schematic side view of the experiment, where the stimulating pipette constituted the known point-current source at a *z*-distance of 7 μm above the electrode array. The pipette was kept at the same *z*-distance during lateral movements of 20 μm parallel to the surface in the *x*–*y* plane. **(B)** Differential interference contrast (DIC) microscopy image of the electrode array showing el4 (11 μm^2^) electrodes and the stimulating pipette. **(C)** Simulation of the signal amplitudes of four electrode sizes (el1: 86 μm^2^; el2: 44 μm^2^; el3: 22 μm^2^; el4: 11 μm^2^) and experimental recordings of signal amplitudes of two electrode sizes (el1: 86 μm^2^; el4: 11 μm^2^), measured while the pipette was moved in the *x*–*y* plane at a *z*-distance of 7 μm above the electrode array in saline. Amplitudes were normalized to the maximum signal amplitudes detected. The inset image shows bright Pt electrodes of four different sizes (el1–el4). **(D)** Simulation of relative detectable signal amplitudes upon increasing the z-distance (*z* = 1, 10, 20, 100 μm^2^) and for varying the electrode size (1 × 1, 2 × 2, 4 × 4, 8 × 8, 16 × 16, 32 × 32, 64 × 64, 100 × 100 μm^2^). The largest obtained signal was set to one.

The electrode size determines, how much charges are detected in a given environment. Larger electrodes detect potentials over a larger area, but they may also contribute to blurring highly local events due to spatial averaging, i.e., the fact that the recorded signal is averaged over a comparably large surface. Spatial averaging not only limits the level of spatial details that can be extracted from neuronal signals, but also affects the peak-signal amplitudes. In a first approach, we quantified the spatial averaging in dependence of the electrode size under controlled conditions by using a stimulating point-current source or micropipette close to the electrode as model signal-source, as shown in Figure 5A,B (Supplementary Figure S1a shows the electric field of the point-current source). By using the micropipette approach, the signal source is well-defined, and we were able to examine parameters, such as the precise location of the signal source and the lateral and vertical distance between electrode and source (Obien et al., 2019). The micropipette (point-current-source) recordings were done for two electrode sizes el1 (86 μm^2^) and el4 (11 μm^2^) and are shown in Figure 5C. The *z*-distance between the stimulation electrode and the array was 7 μm. The recorded signal amplitudes for each electrode size were compared to simulated spatial signal distributions (Figure 5C). For the simulations, a MoI was used (Ness et al., 2015; Obien et al., 2019). The analysis also encompassed much smaller (down to 1 μm × 1 μm) and larger electrodes (up to 100 μm × 100 μm). As shown in Figure 5C,D, spatial-averaging owing to electrode size was highly dependent on the position of the signal source. If the signal source was close to the electrode, either laterally (*x, y*) or vertically (*z*), the averaging effect of larger electrodes was more pronounced. For example, for a signal from a source at *z* = 1 μm a 95% signal amplitude reduction was observed upon using a 100 μm × 100 μm electrode as compared to a 1 μm × 1μm electrode. However, if the signal source was at a *z*-distance of 100 μm, spatial-averaging was almost negligible, so that both electrode sizes, 1 × 1 and 100 μm × 100 μm, provided approximately the same signal amplitude.

Additionally, we analyzed the spatial-averaging effect on the signal peak amplitude with a point source and an electrode array that featured four different electrode sizes (see Figure 1B,C, inset in Figure 5C). To obtain an estimation of the averaging effect, we calculated the slope between the largest signal amplitude at the center electrode and the amplitudes measured 18 μm away (location of the next neighboring electrode in a hexagonal arrangement) versus distance. The obtained results were: el1 | slope| = 0.022 μm^-1^; el2 | slope| = 0.027 μm^-1^; el3 | slope| = 0.031 μm^-1^; el4 | slope| = 0.034 μm^-1^. The slope became steeper with decreasing electrode size, and the averaging effect was reduced (Figure 5C).

We then tested the effects of spatial-averaging, which we had observed with a point-current-source, with neurons. For the experimental recordings, shown in Figure 4A3,B3,B5, the EAP signals feature large variations in spatial distribution and extension so that it was necessary to analyze the spatial averaging effect over a broad electrode-size range (from 100 μm × 100 μm down to less than 10 μm^2^). To experimentally realize a broad electrode-size range with our arrays (18 μm electrode pitch), we employed two approaches: (i) for electrode sizes <100 μm^2^, we fabricated small electrodes on the array (el1 to el4: 86 μm^2^, 44 μm^2^, 22 μm^2^, 11 μm^2^ as shown in Figure 1) and (ii) for electrode sizes >100 μm^2^ we approximated large-electrode behavior through “pseudo-large” electrodes by combining signals detected by sets of several neighboring small electrodes.

**FIGURE 6:**
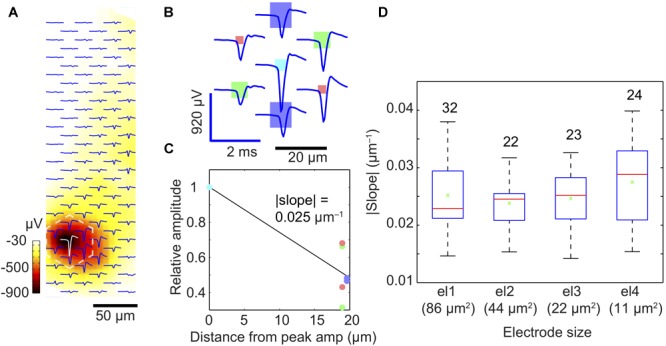
Spatial-averaging of the peak spike amplitude due to electrode size. **(A)** Exemplary extracellular action potential footprint of a neuron in a dissociated-cell culture, recorded by an electrode array with four different electrode sizes (for the electrode size pattern, see **B**). The displayed waveforms represent an average of 500 spontaneous spikes. The color bar (dark red to white) indicates the amplitude of the negative peak signals detected by the electrodes. **(B)** Close-up of the marked area in **(A)** (dashed white lines) to show the individual waveforms recorded by the electrodes of different sizes (displayed to scale as rectangles: dark blue: 86 μm^2^; green: 44 μm^2^; light blue: 22 μm^2^; red: 11 μm^2^). **(C)** Relative amplitudes (relative to the maximum signal at the light blue electrode) recorded at six electrodes surrounding the ‘center electrode’ recording the largest signal per neuronal footprint. The steepness of the slope in **(C)** reflects the spatial-averaging effect of the central electrode; the steeper the slope, the lower is the spatial-averaging of the central electrode according to its size. The color of each dot corresponds to those of the electrodes in plot **(B)**. **(D)** Signal averaging results obtained from 101 neurons and shown as a quartile box plot (red line indicates the median, green cross shows the mean, the box edges represent the quartiles, and the whiskers represent the maximum and minimum slopes obtained). The numbers on top of each box/bar indicate the number of measured units or neurons.

The first method was applied to small electrode sizes (el1 to el4). We recorded EAPs from dissociated cortical cell cultures (DIV 18) of 101 identified units or neurons using an electrode array with four electrode sizes (el1 to el4). Each electrode in that array (hexagonal arrangement) featured a defined size and was surrounded by a total of six electrodes, i.e., two electrodes of each other size, see Figure 1B,C, inset in Figure 5C). Figure 6A shows an electrical-footprint of an identified unit or neuron. We selected the electrode featuring the largest negative spike (perisomatic area) as a central electrode and used the six next neighboring electrodes (total of seven electrodes, Figure 6B) to estimate the spatial-averaging effects in dependence of the electrode size. We had to cope with two effects of neurons that rendered our analysis a bit more difficult, (a) the variation in signal amplitudes across different neurons and (b) the anisotropy of the electrical potential distribution around the neuronal region producing the largest extracellular signal (AIS). By determining a relative peak-signal amplitude of the central electrode with respect to the signals of neighboring electrodes, instead of using absolute signal-amplitude values, we could cope with variations in the signal amplitudes of different neurons, which may arise from neuron physiology, relative position to the electrodes, or morphology. With regard to the second issue, anisotropy, our simulations (Figure 5C) showed that there is no significant difference in signal amplitudes detected by differently sized electrodes far from the peak signal. The differences in relative signal amplitudes for different electrode sizes (el1 to el4) became rather small, as soon as the lateral distance between the signal source and the recording electrode became larger than 18 μm (Figure 5C), which is the array electrode pitch. We noticed, however, that in the experiments the EAP signal amplitudes recorded from the six electrodes surrounding the center electrode (featuring the largest signal) were significantly different under essentially all circumstances (Figure 6C). To some extent this is due to the fact that one cannot expect an isotropic signal distribution around a neuron as, unlike with an ideal point source, the distribution of ion channels across the morphologically anisotropic neuron influences shape and spatial distribution of the EAP signal. Typically, neurons resemble more like a dipole, with the sum of currents being zero. Moreover, a neuron has an axon along which an action potential propagates and an AIS, which usually is the region of largest observable signals (Bakkum et al., 2018). Therefore, electrodes in the vicinity of the axons or AIS will detect larger signals. Additionally, the presence of glial cells will affect the spreading or sealing resistances of the recording electrode. If the electrode is completely covered by a glial cell, the signal detected by the covered electrode will be attenuated. On the other hand, if an axon is on top of an electrode, which is then covered by glial cells, the amplitude of the propagating axonal action potential, detected by the electrode, will be higher due to the ‘amplification’ effect of a resistive layer on top of the signal source (Matsumura et al., 2016; Obien et al., 2019).

We assumed that by pooling and considering the recordings of many neurons we would be able to detect spatial-averaging effects of the electrodes. We, therefore, searched for clearly identifiable neurons/units and determined the electrode featuring the largest signal amplitude (most probably at the location of the AIS) and the six surrounding electrodes. We then calculated an average slope between the signal of the center electrode (largest amplitude set to one) and the corresponding relative signal amplitudes on the six surrounding electrodes (Figure 6B,C). For example, the | slope| was 0.025 μm^-1^ for the light blue electrode of type el3 featuring 22 μm^2^ area in Figure 6C. We then determined such signal-slopes for many detected neurons and the respective central electrodes of different sizes. Figure 6D presents the summary and general trend of signal-slopes for 101 neuronal footprints, with center electrodes of different sizes. Although neurons could not be modeled as an ideal point source, we assumed that the slopes calculated from a large enough number of measurements will help to reveal signal averaging effects of the respective central electrodes. This was confirmed by the findings in Figure 6D. Similar to the trend in the point-source simulation, the median slope (red bar) tends to increase from larger to smaller center electrode size, which indicates that spatial-averaging may reduce signal amplitudes, even for electrode sizes below 10 μm × 10 μm.

**FIGURE 7:**
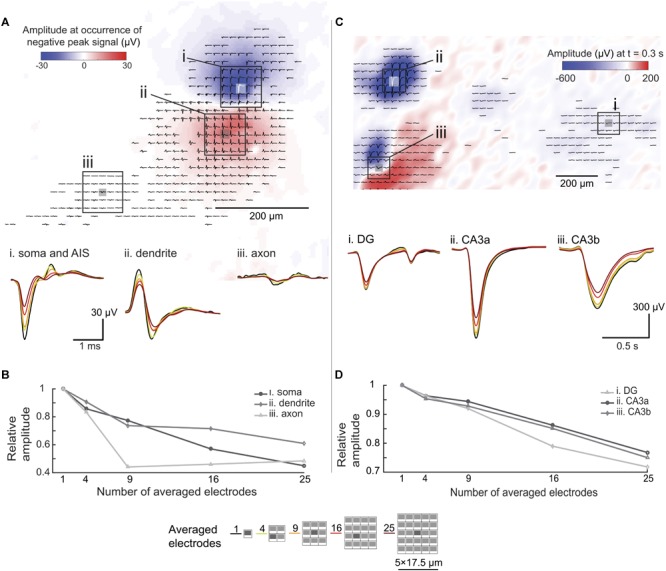
Spatial-averaging of EAPs and LFPs due to electrode size. **(A)** Extracellular action potential (EAP, bandpass-filtered between 300 Hz to 3 kHz) footprint of a hippocampal neuron (organotypic slice, WT mouse, DIV 17) derived from spontaneous activity. The color map (blue to red) indicates the signal amplitude distribution at the occurrence of the largest negative spike peak in the AIS/somatic region. The waveforms (averaged over 500 trials) show different spike shapes indicating different areas of the neuron. Three areas were chosen for signal averaging assessment: (i) the perisomatic area, (ii) the dendritic area, and (iii) an axonal branch. Waveform changes due to the spatial-averaging are shown at the bottom. The color coding refers to the number of averaged electrodes indicated at the bottom of this figure. **(B)** Relative peak-to-peak amplitude differences due to the spatial-averaging effects in different areas of the neuron (electrode configurations used for averaging are shown at the bottom of this figure). **(C)** Local field potential (LFP, bandpass-filtered between 0.1 to 10 Hz) amplitude distribution map, obtained from a single spontaneous event in a hippocampal slice (organotypic culture, WT mouse, DIV 17). The color map (blue to red) indicates the signal amplitude distribution of the LFP 0.3 s after the occurrence of the maximum amplitude signal. The coloring and displayed waveforms (filtered signal detected within 1 s of the recording) indicate different LFP amplitudes and shapes in different areas of the slice. Three areas were chosen for signal spatial-averaging assessment (i) the CA3a area, (ii) the CA3b area, and (iii) the dentate gyrus (DG) area. The displayed waveforms at the bottom show signal alterations upon spatial averaging. Again, the color coding refers to the number of averaged electrodes indicated at the bottom of this figure. **(D)** Relative peak-to-peak amplitude differences due to the spatial-averaging effects in different areas of the slice (electrode configurations used for averaging are shown at the bottom of this figure).

To investigate the spatial-averaging effect on large electrodes, we applied the approach of using pseudo-large-electrodes achieved by averaging the signals detected by sets of multiple neighboring small electrodes. For this analysis, we used an electrical footprint of a hippocampal neuron in an organotypic slice at DIV17, obtained by spike-triggered-averaging over 500 spike events (Figure 7A,B). Previous work with a similar preparation and HD-MEA (Gong et al., 2016), showed the variability in amplitude (∼10 μV) and spread (∼50 μm) of neuronal EAPs across multiple slices over days *in vitro*. We, therefore, considered the electrical footprint shown in Figure 7A as representative of the potential distribution in organotypic slices. We chose regions in the perisomatic area (i), the dendritic area (ii), and in regions of axonal branches (iii) based on spike shapes. To simulate large electrodes, we averaged the signals that had been simultaneously detected by multiple electrodes. The area covered by the averaged electrodes are shown in Figure 7: 4 electrodes: 26.5 μm × 23 μm, 9 electrodes: 44 μm × 40 μm, 16 electrodes: 61.5 μm × 57.5 μm, and 25 electrodes: 79 μm × 75 μm. Each electrode was 9 μm × 5 μm in size, and the electrode pitch was 18 μm. We averaged the waveforms detected by a defined subset of electrodes. This approach gives only a rough approximation of the spatial-averaging effect and is not equivalent to acquiring signals with large contiguous electrodes. Figure 7B summarizes the trend of amplitude reduction through increasing the number of combined electrodes. Signals with high-spatial-frequency components, such as propagating axonal action potentials, suffered more from spatial-averaging effects, compared to large perisomatic signals and signals that cover larger areas, such as dendritic-return-current signals. The results evidenced that the use of smaller electrodes, in this case 9 μm × 5 μm in size, entailed less signal attenuation than using the pseudo-large electrodes.

We applied the same approach to LFP events in an organotypic hippocampal slice (Figure 7C,D). Unlike in the case of EAPs, spatial-averaging had relatively little effect on the LFP signals. As shown in Figure 7D, spatial averaging of the signals of 25 electrodes reduced the amplitude by only approximately 20%, which is of minor relevance in terms of detectability and SNR given the large amplitudes of LFP signals. However, as shown in Figure 7C (iii. CA3b), spatial details of the LFP signals may get lost upon using large electrodes.

### Electrode Density and “Being at the Right Spot”

**FIGURE 8:**
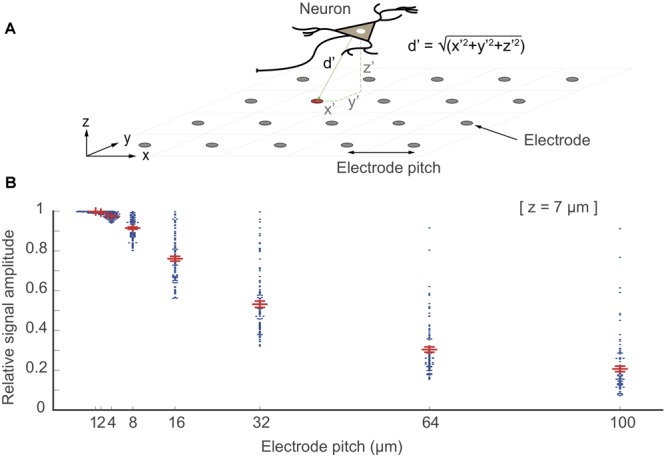
“Electrode being at the right spot”. In this simulation, point electrodes were arranged as an array within 1 × 1 mm^2^ of space at different electrode pitch configurations. 100 point-current sources were placed randomly above the array at a *z*-distance at 7 μm (one neuron as a source is shown in the top figure). The relative signal amplitude, acquired by the electrodes, depended on the distance (*d*′ = √(*x*^′2^+ *y*^′2^+ *z*^′2^) of the electrodes to each of these point-current sources (located at *x*′*y*′z′). The largest signal amplitudes of each given signal source that have been picked up by the electrodes are plotted as points for each electrode-pitch array configuration. The signal amplitudes were normalized with respect to the largest signal amplitude recorded by a given electrode for all configurations.

An important factor that affects signal amplitudes of biological preparations includes the exact location of the neuronal signal source and the probability of an electrode of “being at the right spot.” The probability of “being at the right spot” is directly related to electrode size and density, i.e., the spatial resolution of the electrode array. One can either use large electrode sizes to increase the probability to be close to locations with large signal amplitudes at the expense of increased spatial averaging, or use dense arrays of small electrodes, which will obviate spatial averaging, while there will always be an electrode “at the right spot.” To quantify this effect, we simulated the dense-array scenario by placing point-current-sources at random spatial locations over an array electrode (ideal point electrodes) with different electrode pitches (i.e., 1, 2, 4, 8, 16, 32, 64, 100 μm) as shown in Figure 8A. For analysis, 100 point-current-sources were placed randomly over a 1 mm × 1 mm array area (*x* ×*y*), with a fixed *z*-distance of 7 μm. The largest-signal amplitudes, picked up by the array electrodes for all predefined electrode-pitch values, were plotted as points in Figure 8B. The results show that an electrode array featuring a smaller electrode pitch (<16 μm electrode-pitch; high resolution) could pick up signals from all of the signal sources with minimal attenuation (approximately 20% signal attenuation), as there was always an electrode at the right spot. The chances of getting signals without attenuation is much lower in the case of an array with larger electrode pitch (>32 μm electrode-pitch; low resolution). A point-current source certainly is an oversimplification of a neuron and a dipole is often used to model a neuron. However, for the consideration here, a dipole would typically result in electric potentials with larger local changes, requiring even higher electrode density. Therefore, a single point source, is a good lower bound showing the signal loss due to not having an electrode at the right spot.

Electrode density or “being at the right spot” effect is significant, when signal sources are close to the electrode array or for signals featuring small spatial extension such as axonal signals. From the signal characteristics (Figure 7), we estimated the *z*-distance (or slope) for signals of different neuronal origin (axonal branches, *z* = 5 μm; somatic areas, *z* = 20 μm; dendritic areas, *z* = 30 μm; LFP, *z* > 50 μm) and calculated the effects of electrode density or pitch, see Supplementary Figure S5.

The ratio of cell density and electrode density affects the separability of neuronal units (Jäckel et al., 2012). When the cell density exceeds the electrode density, the spike sorting performance decreases. In case of overlapping neurons, especially in tissue, the respective neuronal EAP distributions can be revealed by using high-density MEAs.

### Readout Circuitry: Signal Attenuation

**FIGURE 9:**
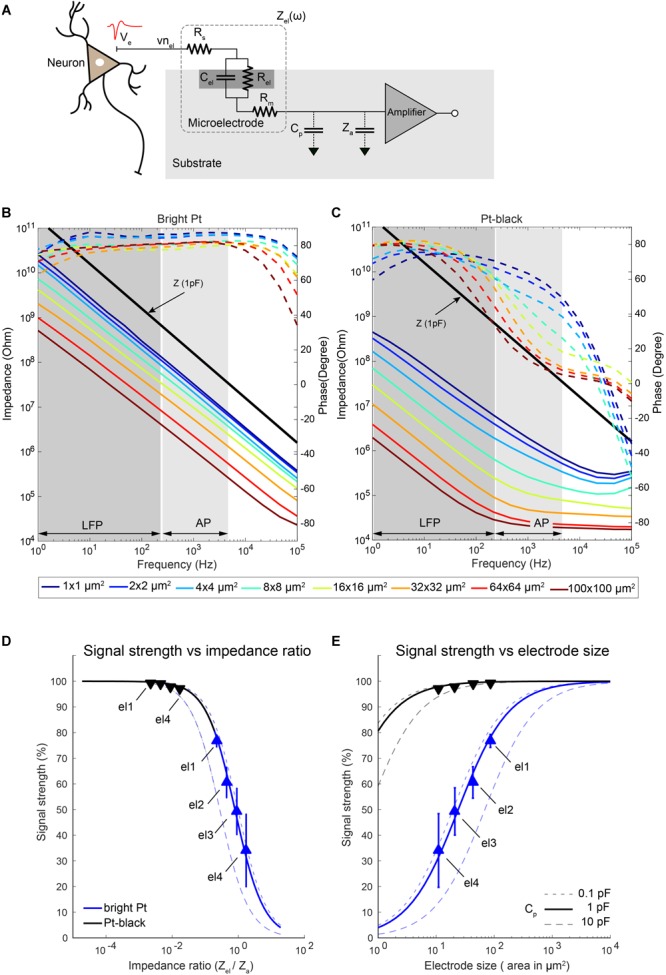
Electrode impedance and effects on signal attenuation. **(A)** Equivalent-circuit model of a metal microelectrode for electrophysiology recordings as adapted from Robinson (1968). **(B,C)** Electrode impedance of electrodes of various sizes as a function of frequency (solid lines: impedance magnitude; dashed lines: impedance phase). Impedance spectra of bright Pt **(B)** and Pt-black-coated **(C)** electrodes are shown. Electrode size varied from 100 μm down to 1 μm diameter. The electrodes were fabricated, and their impedance was measured in a frequency range from 1 Hz to 100 kHz. The input impedance of an amplifier with a capacitance of 1 pF is also plotted here for comparison. **(D,E)** Show the attenuation of the recorded signals through bright Pt and Pt-black electrodes for different ratios of the electrode impedance to the amplifier input impedance **(D)** as well as electrode sizes **(E)** at 1 kHz. The amplifier input capacitance was taken as 3.8 pF (Frey et al., 2010), based on measurements, and the shunt capacitance was swept between 0.1 pF to 10 pF as shown in the figures. The measured signal attenuation due to impedance ratios/electrode sizes is displayed for 4 electrode sizes (el1: 86 μm^2^; el2: 44 μm^2^; el3: 22 μm^2^; el4: 11 μm^2^). Blue lines represent bright Pt electrodes, and black lines represent the Pt-black electrodes. In the bright-Pt case, the smallest electrode (el4: 11 μm^2^) showed a signal-attenuation of over 70%, and the largest electrode (el1: 86 μm^2^) around 30%. By reducing the absolute electrode impedance through Pt-black deposition, the effects of signal-attenuation decreased to <2% for all electrode sizes.

The electrode size (electrode–electrolyte interface area) directly determines the impedance of the electrodes—another factor for signal-attenuation in the signal-recording chain. Figure 9A shows the equivalent-circuit model of an electrophysiology recording channel, from the neurons to the amplifier input, adapted from Robinson (1968) and Obien et al. (2015). The ratio of electrode impedance (*Z*_el_) to amplifier input impedance (*Z*_a_) and the routing (or shunt) capacitance (*C*_p_) attenuates the signal magnitude recorded at the electrode (Nelson et al., 2008).

To check the size-dependence of the signal attenuation, we measured the impedance of electrodes of sizes from 100 μm × 100 μm down to 1 μm × 1 μm as a function of the frequency (Figure 9B,C). Pt-black has been electrodeposited on the electrodes to reduce the impedance, as it effectively increases the electrode–electrolyte interface area while preserving the geometric size. An impedance reduction of >50 times was observed for lower frequencies, especially in the LFP band (Figure 9C). At higher frequencies (AP band), the impedance reduction was limited by the resistive behavior, dominated by the solution resistance (*R*_s_), especially for large Pt-black electrodes (>16 μm × 16 μm). The signal-attenuation as a function of the impedance ratio (*Z*_el_/*Z*_a_) was simulated for different electrodes and then confirmed through measurements (Figure 9D,E). For the simulation, estimated unit capacitances of 0.2 pF/μm^2^ for bright Pt electrodes and 30 pF/μm^2^ for Pt-black electrodes obtained by impedance measurements were used, which matched reported values for large electrodes (Robinson, 1968; Franks et al., 2005).

Since the effective input impedance of the amplifier is mainly dependent on the amplifier configuration, we investigated two common types of amplifier configurations (Supplementary Figure S3). The closed-loop amplifier (Harrison et al., 2003; Frey et al., 2010), as modeled in Supplementary Figure S3b, uses a large input capacitance (*C*_i_) and a small feedback capacitance (*C*_f_) to achieve a high gain, which results in a low effective input impedance *Z*_a_ = 1/ω ∙ (*C*_i_ + *C*_a_). In contrast, the input impedance of an open-loop amplifier (Viswam et al., 2016), as modeled in Supplementary Figure S3c, mainly depends on the input transistor gate capacitance (*C*_a_), which is usually an order of magnitude lower than *C*_i_. For the measurements, a closed-loop type recording amplifier with an input capacitance of 3.8 pF (41 MΩ at 1 kHz) (Frey et al., 2010) was used.

As expected, the ratio of *Z*_el_ to *Z*_a_ played an important role for the amplitude of the recorded signals. We measured the signal attenuation by applying a known signal to the electrolyte solution (PBS) though a Pt reference electrode, and measured the signal amplitudes of the microelectrodes. Four electrode sizes (el1: 86 μm^2^; el2: 44 μm^2^; el3: 22 μm^2^; el4: 11 μm^2^) were used for the measurements. We have observed a significant signal attenuation (68%) for the smallest electrode el4 (bright Pt). This is due to the fact that, for smaller electrodes, the electrode impedance is comparable to the input impedance of the voltage-recording amplifier (Figure 9D). By reducing the absolute electrode impedance through Pt-black deposition, the signal-attenuation effect was reduced to <2% for all four different electrode sizes. For the case of the simulated 1 μm × 1 μm electrode, the signal-attenuation was reduced significantly from >95% to <20% after Pt-black deposition. We observed that the signal-attenuation was below 5%, if the electrode impedance was 20 times lower than the amplifier input impedance. In the measurement setup, the parasitic capacitance (*C*_p_) was estimated to be 0.5 pF (Obien et al., 2015). To see the effects of the parasitic capacitance on signal-attenuation, *C*_p_ was swept (from 0.1 to 10 pF) while keeping the same amplifier input impedance (3.8 pF). A lower impedance ratio (realized either by Pt-black deposition or by using larger electrodes) was necessary to cope with higher parasitic capacitances.

### Noise and Signal-to-Noise Characteristics

Noise in extracellular recording refers to all signal contributions that interfere with the neuronal signal of interest. Three main types of noise affect the signals recorded by extracellular electrodes: (1) the inherent thermal noise of the electrodes; (2) the “background activity,” which consists of the background electrical signals of more distant neurons that cannot be identified; and (3) the noise of the recording amplifiers. The quality of extracellular recordings and their SNR depend on how well the signal of interest can be acquired in the presence of noise from the various sources in the recording chain.

#### Thermal Noise

**FIGURE 10:**
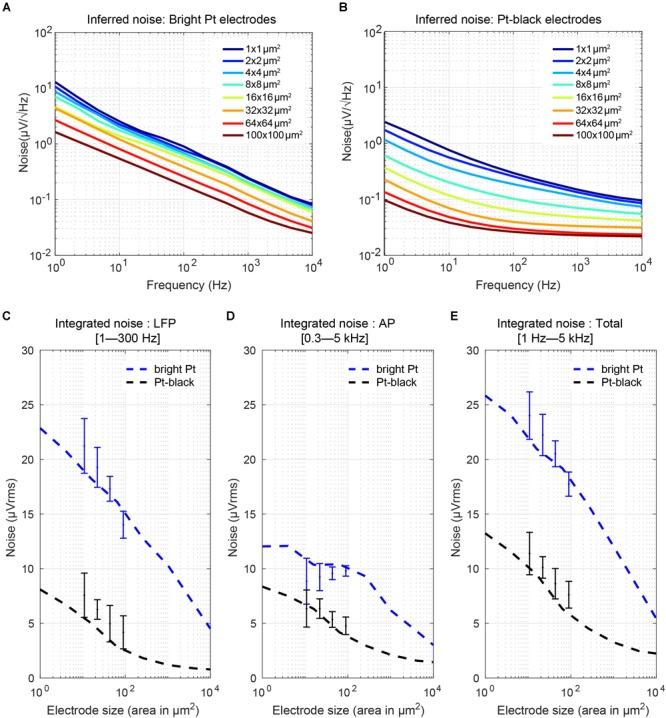
Electrode noise: **(A,B)** Noise power spectral density (PSD) of Pt-electrodes and Pt-black electrodes computed from the real part of the measured electrode impedance. The electrodes show 1/f noise characteristics at low frequencies and reach a plateau of thermal noise at higher frequencies. After Pt-black deposition, the noise spectral density was significantly reduced and reached a plateau defined by the spreading resistance (which depends on the electrode geometric area and solution resistance). **(C–E)** Integrated noise (μV_rms_) of the electrodes in the AP region, LFP region, and over the full bandwidth (1 Hz to 5 kHz) versus electrode size and comparison to measurement results obtained with four electrode sizes (el1: 86 μm^2^; el2: 44 μm^2^; el3: 22 μm^2^; el4: 11 μm^2^) on an HD-MEA. For bright Pt electrodes, the noise in the LFP band increased from 5 μV_rms_ to 23 μV_rms_ for going from a 100 × 100 μm^2^ size electrode to a 1 × 1 μm^2^ size electrode. In the AP band, the noise increase was less, only from 3 μV_rms_ to 13 μV_rms_. After Pt-black deposition, the LFP noise and AP noise was below 8 μV_rms_ for all electrode sizes.

Thermal noise gets introduced to the recorded signal at each electrode. The real part of the impedance is the major contributor to thermal noise, and decreasing the size of microelectrodes results in an increase in their impedance (Robinson, 1968). The equivalent thermal noise can be calculated as follows:

(4) $$\begin{array}{c} v_{n}=\sqrt{4kTRe(Z_{e}^{'})\cdot \Delta f} \end{array}$$

where *k* is the Boltzmann constant, *T* is the absolute temperature, *R*e(*Z*e′) is the real part of the effective electrode impedance, and Δ*f* is the noise bandwidth (Obien et al., 2015). We calculated the real part of the impedance from the measured impedance magnitude and phase for all electrode sizes (Figure 9B,C). The equivalent noise power spectral density (PSD) was estimated (Figure 10A,B) according to the real part of the impedance using Eq. (4) (Sharma et al., 2017). For both, bright Pt and Pt-black electrodes, the PSD shows 1/*f* noise characteristics at low frequencies and reaches a plateau of thermal noise level (dominated by *R*_s_-noise as expected) at higher frequencies. The RMS noise in the LFP band (from 1 Hz to 300 kHz), EAP band (from 300 Hz to 5 kHz) and also the full signal band (from 1 Hz to 5 kHz) was integrated from the noise PSD (Figure 10C–E). We also measured noise values for the four electrode sizes (el1: 86 μm^2^; el2: 44 μm^2^; el3: 22 μm^2^; el4: 11 μm^2^) using the HD-MEA amplification circuits. The measured results showed a good match with the noise values inferred from the electrode impedances (Figure 10C–E). The noise of bright Pt electrodes was generally high across all frequency bands, especially in the LFP band. After Pt-black deposition, the LFP band noise and AP band noise were below 8 μVrms, for all electrode sizes. An initially low electrode impedance helps to reduce the electrode-size dependence of the noise, which turned out to be extremely important for LFP-signal recordings. As mentioned in the previous subsection, low electrode impedance for small geometric electrode sizes can be achieved through electrode surface coating, which increases the surface area while preserving a small geometric electrode area. Coating materials include, e.g., Pt-black or poly-3,4-ethylendioxythiophen (PEDOT) (Kim et al., 2014; Boehler et al., 2015).

#### Background Activity

“Noise” originating from background neuronal activity has to be considered separately for EAP spike detection and LFP signal extraction. For EAP spike detection, background activity comprises the undesired EAPs from distant neuronal sources (>100 μm away from the recording electrode) as well as low-frequency population-activity signals (LFP). The size of the electrodes determines how much background activity gets picked up (Camuñas-Mesa and Quiroga, 2013). Small electrodes record from only a few nearby neurons, so that background activity contributions are low. Moreover, small electrodes offer excellent single-unit isolation capabilities when they are located very close to the neurons of interest. In contrast, large electrodes pick up the “background activity” of more distant neurons within a larger perimeter. A smaller ratio of the signal amplitudes of the nearby neuronal signals of interest to those of the more distant cells that contribute to the background activity leads to a lower SNR for individual electrodes (Harris et al., 2016).

For LFP signal extraction, all neuronal activities at high frequencies (AP spikes) are undesired and can be considered as a background activity. Although the signal can be specifically filtered for the LFP band of interest, the effect of AP bleed-through on the signal remains and may cause some noise (Ray, 2015). Additional techniques need to be employed to remove the spike-related transients in LFP-filtered signals, e.g., the subtraction of the mean spike waveform from the wide-band signal before low-pass filtering (Pesaran et al., 2002) or the interpolation of the LFP signal in a pre-determined interval before and after the spike (Okun et al., 2010). Supplementary Figure S6 shows the characteristics of background activity for different electrodes sizes in a cell culture measurement. The background activity is given in μV_rms_ (root mean square) including spiking activity (2,400 data points per electrode type) and with exclusion of electrodes that detected spikes (1,570 data points per electrode type). The measurements showed that the smallest electrodes picked up slightly less “background activity,” while they featured higher intrinsic thermal noise. The intrinsic thermal noise component for each electrode size and the electronic noise of the amplifiers has been subtracted in the results displayed in the Supplementary Figure S6. Background activity values heavily depend on preparations, cell types, number of active neurons and other culture or preparation parameters, e.g., temperature, etc.

#### Noise From Recording Amplifiers

Amplifier noise also deteriorates the SNR of extracellular recordings but does not scale with electrode size. By careful design of the recording amplifiers, the amplifier noise can be kept well below the noise generated by the microelectrodes. This aspect becomes very important for designing amplifiers for HD-MEAs, where power-consumption and circuit-area limitations need to be considered (Obien et al., 2015). In general, it is difficult to design small-footprint amplifiers with very low noise.

#### SNR in Dependence of Electrode Size

We estimated SNRs considering all effects described above that depend on electrode size: (1) the spatial-averaging effect; (2) the effect of being at the right spot; (3) the signal-attenuation due to the impedance ratio (*Z*_el_/*Z*_a_); and (4) the electrode noise – including both, thermal noise and background activity.

Taking into account all these parameters we tried to determine optimal electrode sizes for EAP and LFP recordings. To calculate the corresponding SNR values (Eq. 3), the mean signal amplitudes (EAP and LFP) of individual neurons and collective activity, identified in the recordings, were used (see Figure 7A–D). Then, the spatial-averaging effect, the effect of being at the right spot and signal-attenuation characteristics were taken into account for the different electrode sizes, and the obtained values were divided by the noise values (including both, thermal and background activity) for each electrode size.

**FIGURE 11:**
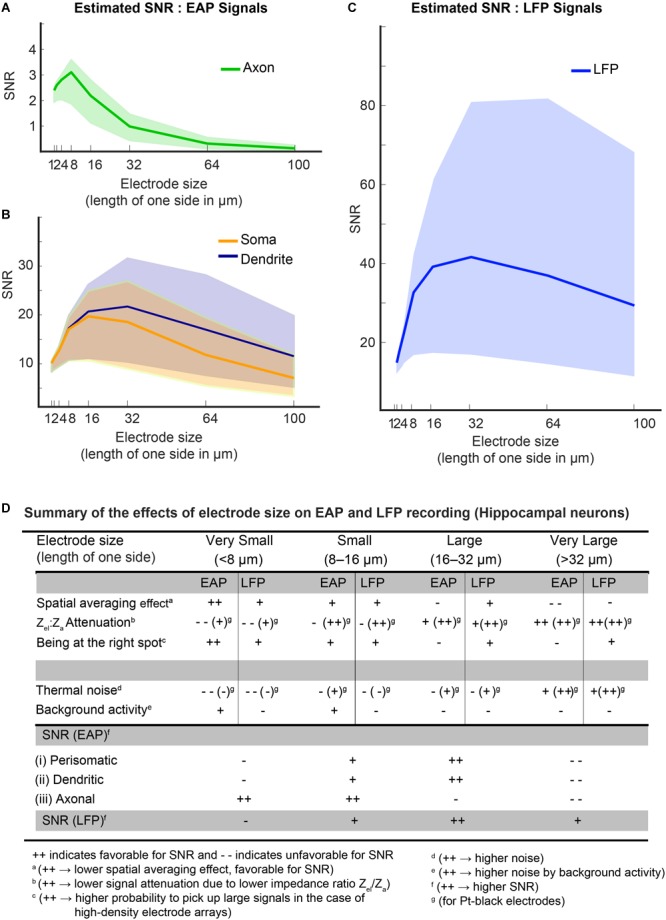
**(A–C)** Estimated SNRs for EAP and LFP signals from hippocampal neurons. To estimate the SNR, the dependence of the main parameters on electrode size was considered: spatial-averaging effect; the effect of being at the right spot; signal-attenuation due to the impedance ratio (*Z*_el_/*Z*_a_); and noise – both thermal noise of electrode and background activity. The background activity level played a pivotal role in determining the optimal electrode size in both, the EAP and LFP band. Colored bands indicate the SNR levels at different levels of background activity (lower bound of the band is for 8 μV_rms_, upper bound at 1 μV_rms_ and the median at 3 μV_rms_). For an axonal branch **(A)**, small electrodes (size < 16 × 16 μm) show peak SNRs. But for somatic or dendritic signals **(B)**, a medium-size electrode (size 8 × 8 μm^2^ – 30 × 30 μm^2^) were observed to be optimal. LFP signals generally feature high SNRs **(C)**, and electrodes down to 16 × 16 μm^2^ size provide good SNR for all LFP signals. **(D)** The table summarizes the effects of electrode size on EAP and LFP recording for hippocampal neurons.

The colored bands in Figure 11A–C show the estimated SNRs for both, EAP and LFP signals from organotypic hippocampal slices for different electrode sizes. The figures demonstrate that the background-activity level (low: 1 μV_rms_, medium: 3 μV_rms_ and high: 8 μV_rms_; Camuñas-Mesa and Quiroga, 2013) plays a pivotal role in determining optimal electrode size ranges for each signal type. For low-amplitude axonal-branch signals, achieving a good SNR is critical, so that smaller electrode sizes (<16 μm × 16 μm) should be used to obtain SNR-values > 2, especially when there is high “background activity” (Figure 11A). For high-amplitude signals, like somatic or dendritic signals (SNR > 10), a medium-size (8 μm × 8 μm – 32 μm × 32 μm) electrode seems to be optimal (Figure 11B). As shown in Figure 11C, a large electrode size (>16 μm × 16 μm) was found to be optimal for all types LFP signals, while it has to be noted that the SNR is comparably high over the whole electrode size range for LFPs.

## Discussion

We have shown, through experimental and computational analysis, how the electrode size can influence the signal quality of extracellular recordings. We investigated electrode-size-dependent signal-attenuation, caused by spatial-averaging, and the influence of the interface circuitry. We characterized noise interference, which included both, thermal noise and background electrical activity of other cells. We quantified these effects separately in order to analyze, which effect dominates under which circumstances.

We used modeling and experiments to quantify the effect of spatial averaging of the signals as a consequence of the electrode size. Using the point-current-source approach, we found that the spatial-averaging effect was correlated to the distance between electrode and signal source. For example, for a *z*-distance of 20 μm, electrode sizes below 20 μm × 20 μm did not show any significant differences in the detected signal amplitudes, whereas the signals recorded from electrodes larger than 20 μm × 20 μm were affected by spatial-averaging effects and showed lower signal amplitudes.

We also estimated the effect of spatial-averaging depending on the electrode size for the recorded neuronal signals. Using HD-MEAs, we implemented an experimental approach to reveal spatial-averaging-effect trends for EAP and LFP recordings. Propagating axonal action potentials produce small, local signals, which were clearly detectable by using small HD-MEA electrodes but became hard to record for using larger electrodes. EAPs with larger amplitudes, originating from the perisomatic area, and return currents (usually found in the dendritic area) were also affected by spatial averaging. This finding is in agreement with a review reporting that small electrodes (diameter < 20 μm) should be used to detect and distinguish EAPs of nearby neurons (Harris et al., 2016).

Local field potential signals, in contrast, feature a large spatial spread and are less affected by electrode size spatial averaging. Large electrodes can be advantageous for LFP recording due to the fact that noise gets averaged out, as has been stated previously (Harris et al., 2016). However, as can be seen in Figure 7C,D, spatial details may get lost, which are important for determining current-source densities (CSD) (Pettersen et al., 2010; Einevoll et al., 2013; Łęski et al., 2013) and for understanding the source of LFP signals (Buzsáki et al., 2012; Einevoll et al., 2013). Due to the large amplitudes of LFPs, electrodes of any size within the range of the spatial-signal spread can detect the signal, and the quality of the recordings will be predominantly affected by noise.

Another effect that we analyzed was the electrode-size dependent signal-attenuation through interface circuitry. We observed that an increase in the electrode impedance by reducing the electrode size causes signal attenuation as a consequence of the voltage division between the electrode impedance, the amplifier input impedance, and the routing capacitances (Nelson et al., 2008). Small electrodes (with diameters of less than 5–10 μm) have high impedance and require amplifiers with very high input impedance, which is usually hard to realize in experimental setups. Instrumentation-dependent signal-attenuation is an important parameter for electrophysiological measurements; an impedance ratio of *Z*_el_/*Z*_a_ < 0.1 needs to be established, e.g., by applying surface-modification techniques like Pt-black deposition, to achieve an optimal electrode-amplifier-interface matching.

We then quantified the effect of electrode size on noise, since it directly determines the SNR. Our results showed that electrode impedance reduction (e.g., through Pt-black deposition) was of pivotal importance to achieve low noise values, in both, EAP and LFP signal bands. After Pt-black deposition, the LFP noise and EAP noise (inferred from the electrode impedance) were below 6 μV_rms_ and 8 μV_rms_ for all electrode sizes (1 μm × 1 μm – 100 μm × 100 μm). In the LFP band, the inherent thermal noise of the electrodes was lower than the measured noise. Pt electrodes are polarizable with very small Faradaic currents, so that noise from other sources, e.g., 1/*f*^2^ -noise also needs to be included at frequencies below <10 Hz (Hassibi et al., 2004).

We have also taken into account the background electrical activity of more distant neurons or within neuronal networks. The values for background activity were based on previous work, where background activity simulations using compartmental models were performed (Camuñas-Mesa and Quiroga, 2013). 1 mm^3^ volume of neurons was reported to be a good estimate for hippocampal recordings. The effect of background activity was found to increase, as the number of active neurons increased, for example, from 1.5 μV_rms_ for 2% active neurons to 3.5 μV_rms_ for 14% active neurons within a 1 mm^3^ volume. We utilized 1, 3, and 8 μV_rms_ background-activity levels in estimating the SNR for different electrode sizes.

Finally, we combined all data (spatial-averaging, the effect of being at the right spot, signal-attenuation, electrode noise, and background activity) to obtain SNR approximations for different signal types and electrode sizes (Figure 11). Based on computational work (Camuñas-Mesa and Quiroga, 2013), Camuñas-Mesa and Quiroga (2013) found that SNR was optimal for electrode diameters between 30 μm and 50 μm, and they chose 40 μm as the optimum size for spike-sorting of EAPs recorded from the hippocampus *in vivo*. Electrode diameters smaller than 20 μm were not analyzed, and LFPs were also not considered. According to our results, the optimum electrode size highly depends on the nature of the signal, the signal-source position relative to the electrodes, and the level of background activity. For all electrode sizes, SNR values were found to be high (SNR > 10), when the background activity was in the range of 1–3 μV_rms_. Therefore, we focused on finding an optimum electrode size for higher background-activity levels, where the electrode size becomes important. For small and localized axonal signals, the best SNR was observed for electrode sizes between 1 μm × 1 μm – 16 μm × 16 μm, where the range between 1 μm × 1 μm – 8 μm × 8 μm turned out to be optimal. For large perisomatic spikes and return currents in the dendritic area, the optimum electrode size depends on the background activity levels, with the 8 μm × 8 μm – 32 μm × 32 μm size range being optimal for high background activity. Overall, the optimum electrode-size range for EAP recordings was determined to be 1 μm × 1 μm – 16 μm × 16 μm for axonal signal detection, and 8 μm × 8 μm – 32 μm × 32 μm for large somatic/AIS spike detection. For LFP recordings, SNR values were found to be larger for electrode sizes >16 μm × 16 μm, while smaller electrodes still featured relatively high SNRs, since LFP signals generally have a large amplitude.

We quantified by simulations another factor that directly affects the resolution of electrode arrays (especially for low spatial-extension EAP signals)—the probability of an electrode being located at the “right spot.” According to Camuñas-Mesa and Quiroga (2013), large electrodes have a higher probability of being physically near neuronal sources and of picking up higher-amplitude spikes. Previous studies, such as Moxon (1999), Paik et al. (2003), Ward et al. (2009), Andersen et al. (2010), claim that large recording electrodes can record from more neurons simultaneously. However, large electrodes come at the expense of an averaging effect, which lowers signal peak amplitudes. HD-MEAs include thousands of small electrodes at high spatial resolution, and there is no need to enlarge the electrode to be close to the location with the largest signal, as there will always be an array electrode at the “right spot.” We show that in arrays with a large electrode density (<20 μm electrode-pitch) there is a high chance that an electrode is physically located near individual neuronal signal sources so that localized axonal spike events or low-amplitude spikes of, e.g., EAPs from human iPSC-derived neurons (Wainger et al., 2014; Woodard et al., 2014; Amin et al., 2016) can be detected. However, reducing the electrode pitch below 4 μm does not lead to any further improvement in terms of being at the “right spot.”

Finally, the spatially highly resolved information, provided by HD-MEAs, facilitates localization and classification of neurons and significantly improves unit identification and spike sorting (Einevoll et al., 2012; Franke et al., 2012; Jäckel et al., 2012; Harris et al., 2016; Rossant et al., 2016; Radivojevic et al., 2017; Diggelmann et al., 2018), since large electrodes miss local spatial details of extracellular signals (Ruz et al., 2014). Moreover, HD-MEAs with small electrodes can capture sub-cellular features of neuronal signals and have been used to electrically image the propagation of axonal action potentials (Bakkum et al., 2013, 2018; Radivojevic et al., 2017), which would have been averaged out, if large electrodes had been used. In the case of LFP recordings, high-resolution spatial information may help to elucidate the source and propagation of LFP signals, especially when LFPs are recorded simultaneously with EAPs. Figure 11D summarizes the effects of electrode size on EAP and LFP recordings.

## Ethics Statement

All use of animals and all experimental protocols were approved by the Basel Stadt veterinary office according to Swiss federal laws on animal welfare.

## Author Contributions

VV, MO, UF, FF, and AH designed the experiments. VV and MO performed the experiments and data analysis, and wrote the manuscript. UF, FF, and AH reviewed the manuscript and supervised the project. All authors approved the final version of the manuscript.

## Conflict of Interest Statement

MO and UF are co-founders of MaxWell Biosystems AG, which commercializes HD-MEA technology. The remaining authors declare that the research was conducted in the absence of any commercial or financial relationships that could be construed as a potential conflict of interest.
