# Supplementary material for: Optimal Electrode Size for Multi-Scale Extracellular-Potential Recording From Neuronal Assemblies
Source: Front Neurosci. 2019 Apr 26;13:385. doi: 10.3389/fnins.2019.00385 (PMC6498989; doi:10.3389/fnins.2019.00385)
Supplement: Supplementary file 1 [file Data_Sheet_1.pdf]

Supplementary material:

Optimal electrode size for multi-scale extracellular-potential recording from neuronal assemblies

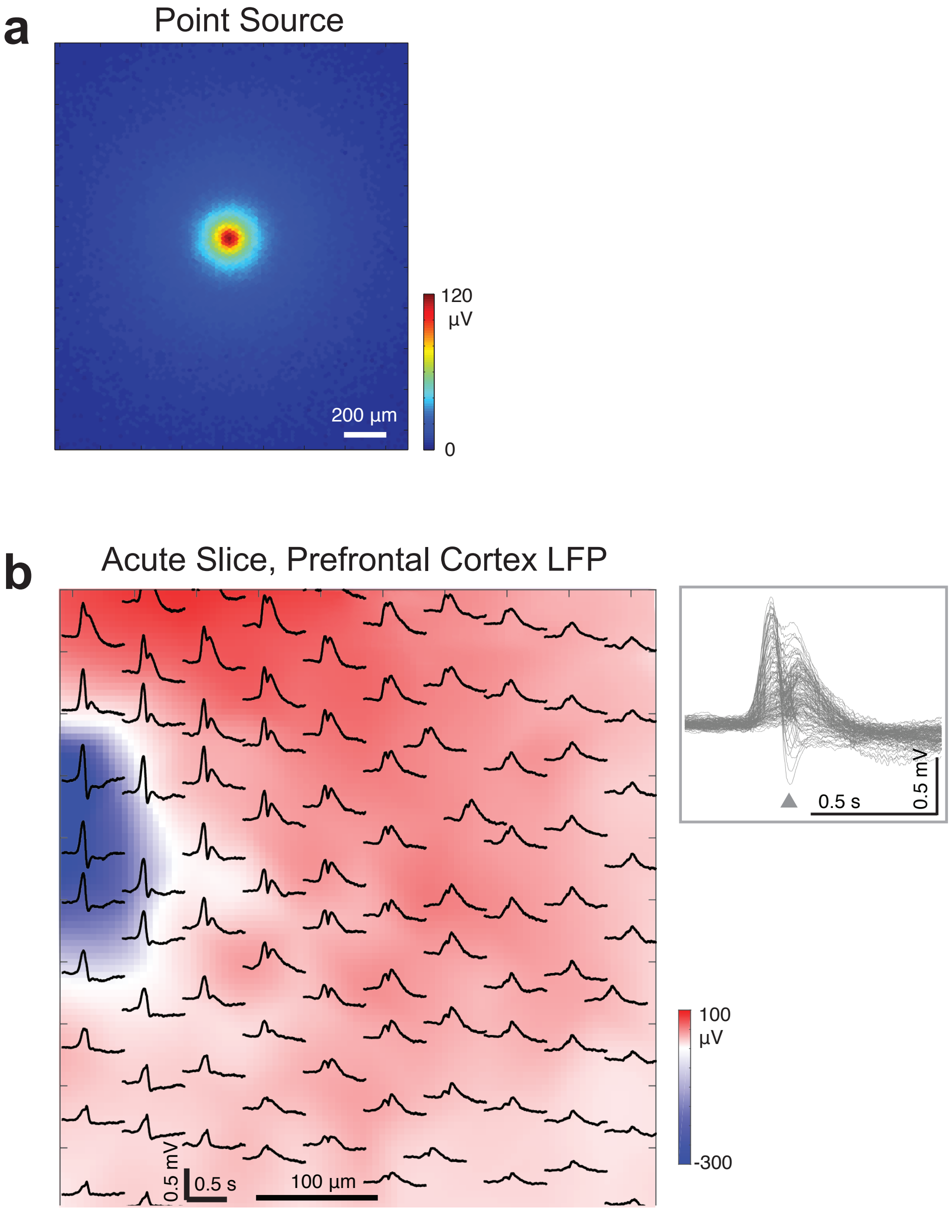

Figure S1. **Signal sources. a**) Stimulation through a pipette as point current source. **b**) Spatio-temporal features of local field potentials, recorded from acute prefrontal cortical slice preparations. The inset shows all the waveforms; the gray triangle marks the time point used for obtaining the color map. One LFP event from an acute prefrontal cortical slice recording (bandpass filter: 0.1-50 Hz).

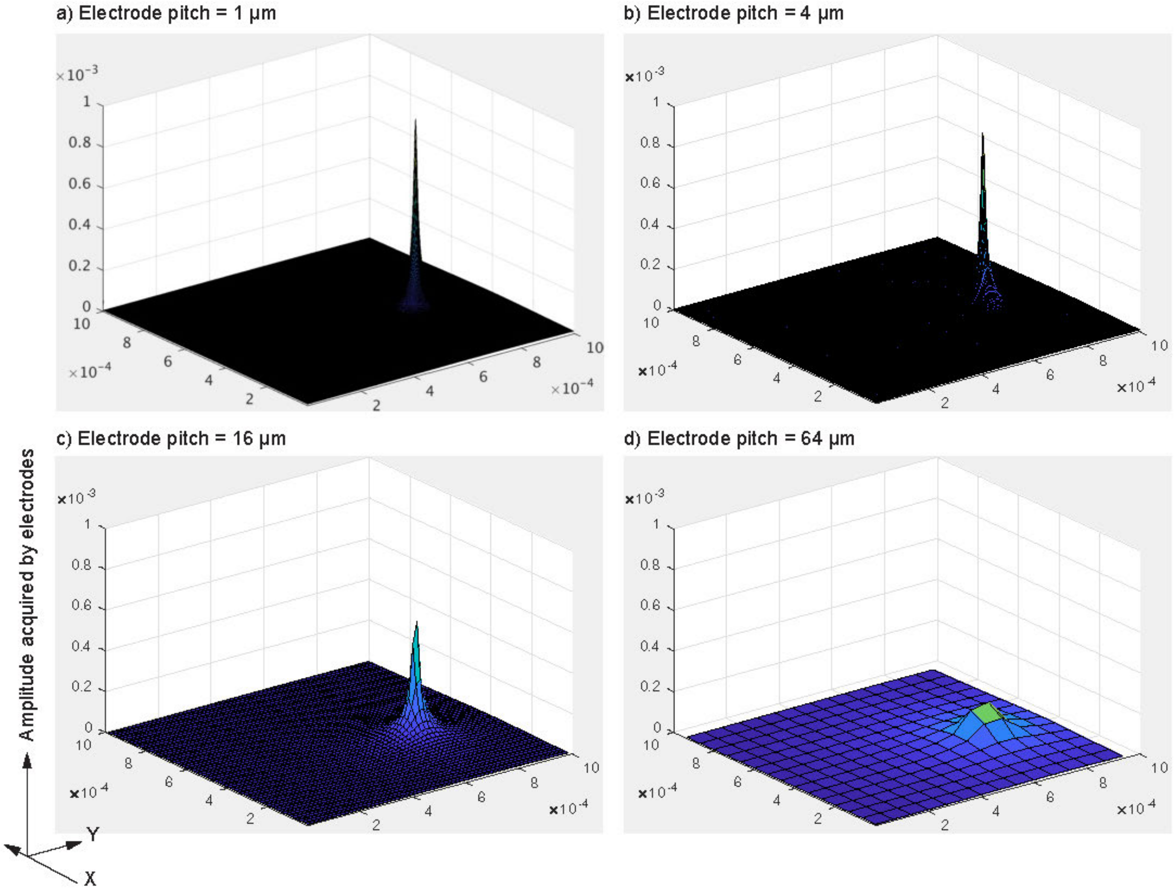

Figure S2. Example plot combining signal-averaging effect. A point-current-source was randomly placed (x' = 392.8 µm, y' = 706.3 µm and z' = 5.462 µm) over an array of uniform electrodes at different electrode pitches. In each configuration, the side length of the square-shape electrode was identical with the electrode pitch, and the electrodes were arranged as a grid (in a 1 × 1 mm^2 area). The surface plots show the signal amplitudes picked by the electrodes at different electrode pitches — **a**) 1 µm **b**) 4 µm **c**) 16 µm **d**) 64 µm. Y-axis shows the amplitude in Volt.

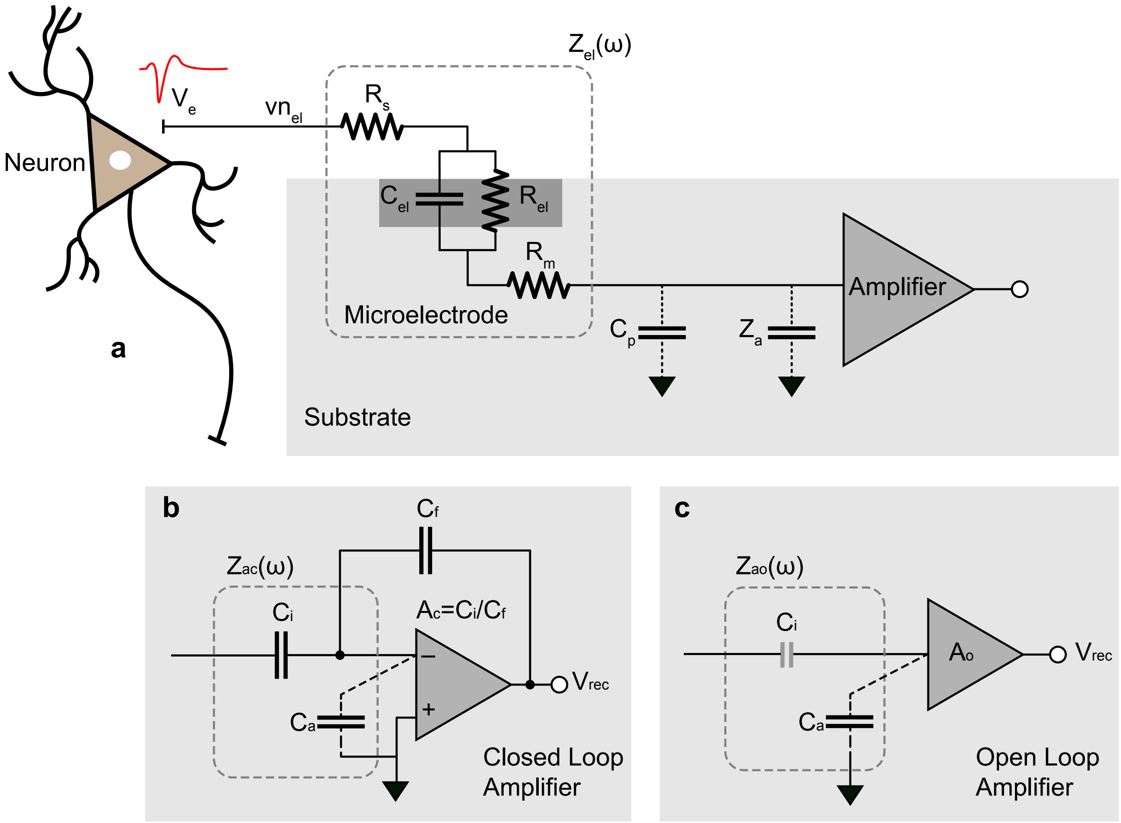

Figure S3. **Equivalent circuit of the microelectrode and recording circuitry channels** (Robinson 1968; Obien et al. 2015). **a**) Equivalent-circuit model of a metal microelectrode for electrophysiology recordings as adapted from Robinson 1968. The circuit includes the electrode in contact with the neurons and the amplifier with a filter. The effective impedance of the electrode (Z_e) includes the resistance of the electrolyte solution (R_s), the resistance and capacitance at the electrode/electrolyte double-layer interface (R_e and C_e), and the Ohmic resistance of the metal electrode (R_m). Lengthy metal leads from the electrode to the input of the amplifier create parasitic capacitances (C_p) and shunt resistances (negligible) to ground. The effective input impedance of the amplifier (Z_a) depends on the amplifier configuration. The triangle represents an ideal amplifier that does not draw any current. The non-ideal aspects of the amplifier have been accounted for in Z_a. The effective input impedance of the amplifier (Z_a) in a closed-loop configuration (**b**) and in an open-loop configuration (**c**).

a) Average distance of a point in a unit “square” from the electrode

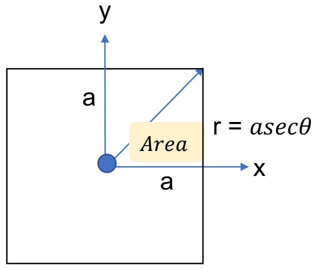

$$d'_{avg} = \frac{8}{4a^2}\int_0^{\frac{\pi}{4}}\int_0^{asec\theta} r^2 dr d\theta$$

b) Grid arrangement

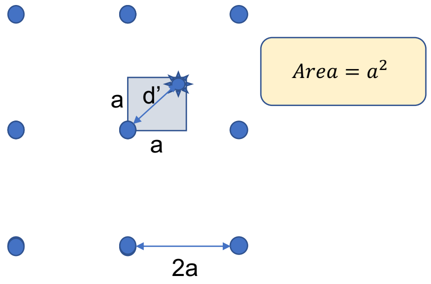

$$d^{G'}_{avg} = \frac{8}{4a^2}\int_0^{\frac{\pi}{4}}\int_0^{asec\theta} r^2 dr d\theta$$

$$= \frac{2}{3a^2}\int_0^{\frac{\pi}{4}} a^3 sec^3\theta d\theta$$

$$= \frac{a}{3}\left(\sqrt{3} + \ln\left(\sqrt{2}+1\right)\right)$$

c) Hexagonal arrangement

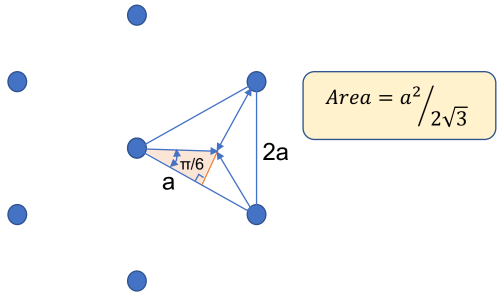

$$d^{H'}_{avg} = \frac{1}{\left(a^2/2\sqrt{3}\right)}\int_0^{\frac{\pi}{6}}\int_0^{asec\theta} r^2 dr d\theta$$

$$= \frac{a}{2\sqrt{3}}\int_0^{\frac{\pi}{6}} a^3 sec^3\theta d\theta$$

$$= \frac{a}{\sqrt{3}}\left(\frac{2}{3} + \ln\left(\sqrt{3}\right)\right)$$

Figure S4. Calculation of the average distance of a point from an electrode. (a) Average distance of a point in a unit square (lateral distance a, origin at the center of the square) in polar coordinates; (b) average distance ($d^{G'}_{avg}$) when the electrodes are arranged in a regular grid, here 2a is the electrode to electrode distance or pitch; (c) average distance ($d^{H'}_{avg}$) when the electrodes are in a hexagonal arrangement.

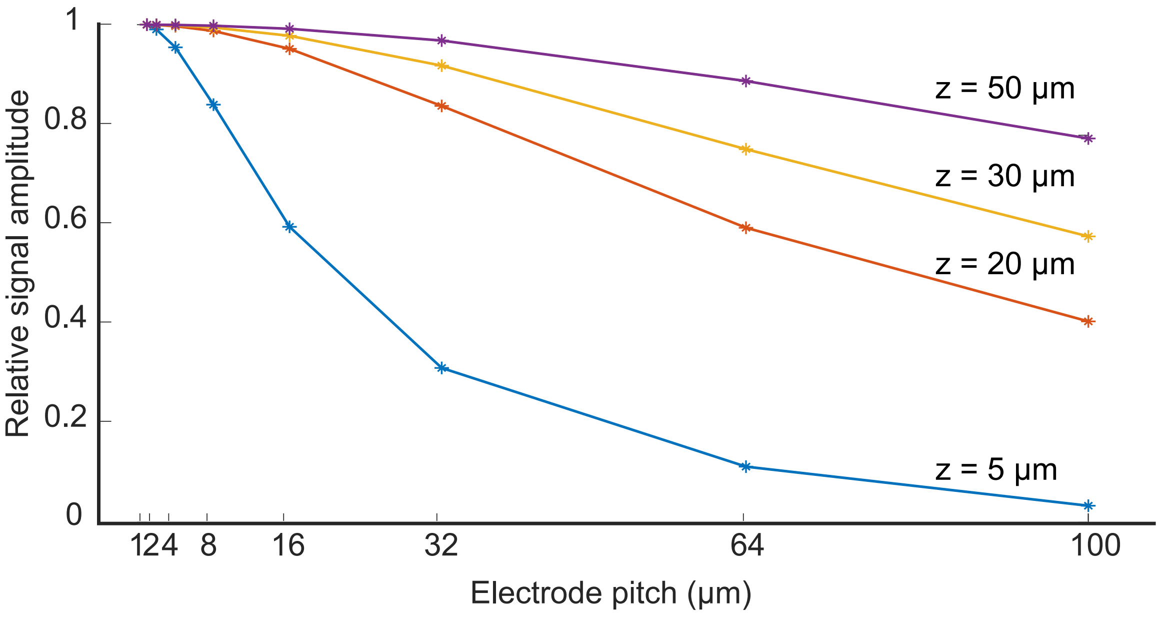

Figure S5. Effects of electrode density or electrode "being at the right spot" simulated for different z-distances. Estimated z-distance (or slope) of each signal type – for axonal branches, z = 5 μm; somatic areas, z = 20 μm; dendritic areas, z = 30 μm and for LFP, z > 50 μm.

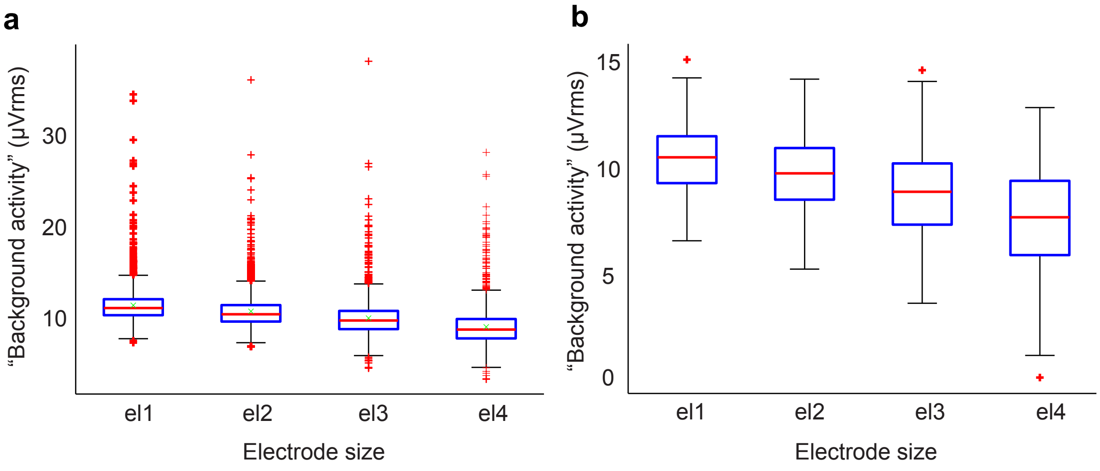

Figure S6. **Background electrical activity a**) Recorded signal activity of a cortical cell culture with AP spikes (2400 electrodes per each electrode size). **b**) Background electrical activity excluding the electrodes with detectable spikes (1570 electrodes per electrode size). The trend in both cases is that smaller electrodes record less background activity (Camuñas-Mesa and Quiroga 2013), which is in contrast to their increased thermal noise. The values here may change depending on culture preparation, cell type, number of active neurons and physical parameters, including temperature etc.
